# L-cysteine methyl ester overcomes the deleterious effects of morphine on ventilatory parameters and arterial blood-gas chemistry in unanesthetized rats

**DOI:** 10.3389/fphar.2022.968378

**Published:** 2022-09-28

**Authors:** Paulina M. Getsy, Santhosh M. Baby, Walter J. May, James N. Bates, Christopher R. Ellis, Michael G. Feasel, Christopher G. Wilson, Tristan H. J. Lewis, Benjamin Gaston, Yee-Hsee Hsieh, Stephen J. Lewis

**Affiliations:** ^1^ Department of Pediatrics, Case Western Reserve University, Cleveland, OH, United States; ^2^ Galleon Pharmaceuticals, Inc., Horsham, PA, United States; ^3^ Pediatric Respiratory Medicine, University of Virginia School of Medicine, Charlottesville, VA, United States; ^4^ Department of Anesthesiology, University of Iowa Hospitals and Clinics, Iowa City, IA, United States; ^5^ United States Army CCDC Chemical Biological Center, Aberdeen Proving Ground, MD, United States; ^6^ Department of Basic Sciences, Division of Physiology, School of Medicine, Loma Linda University, Loma Linda, CA, United States; ^7^ Herman B Wells Center for Pediatric Research, Indiana University School of Medicine, Indianapolis, IN, United States; ^8^ Division of Pulmonary, Critical Care and Sleep Medicine, Case Western Reserve University, Cleveland, OH, United States; ^9^ Department of Pharmacology, Case Western Reserve University, Cleveland, OH, United States

**Keywords:** L-cysteine methylester, morphine, ventilatory depression, arterial blood-gas chemistry, Sprague Dawley rats

## Abstract

We are developing a series of thiolesters that produce an immediate and sustained reversal of the deleterious effects of opioids, such as morphine and fentanyl, on ventilation without diminishing the antinociceptive effects of these opioids. We report here the effects of systemic injections of L-cysteine methyl ester (L-CYSme) on morphine-induced changes in ventilatory parameters, arterial-blood gas (ABG) chemistry (pH, pCO_2_, pO_2_, sO_2_), Alveolar-arterial (A-a) gradient (i.e., the index of alveolar gas-exchange within the lungs), and antinociception in unanesthetized Sprague Dawley rats. The administration of morphine (10 mg/kg, IV) produced a series of deleterious effects on ventilatory parameters, including sustained decreases in tidal volume, minute ventilation, inspiratory drive and peak inspiratory flow that were accompanied by a sustained increase in end inspiratory pause. A single injection of L-CYSme (500 μmol/kg, IV) produced a rapid and long-lasting reversal of the deleterious effects of morphine on ventilatory parameters, and a second injection of L-CYSme (500 μmol/kg, IV) elicited pronounced increases in ventilatory parameters, such as minute ventilation, to values well above pre-morphine levels. L-CYSme (250 or 500 μmol/kg, IV) also produced an immediate and sustained reversal of the deleterious effects of morphine (10 mg/kg, IV) on arterial blood pH, pCO_2_, pO_2_, sO_2_ and A-a gradient, whereas L-cysteine (500 μmol/kg, IV) itself was inactive. L-CYSme (500 μmol/kg, IV) did not appear to modulate the sedative effects of morphine as measured by righting reflex times, but did diminish the duration, however, not the magnitude of the antinociceptive actions of morphine (5 or 10 mg/kg, IV) as determined in tail-flick latency and hindpaw-withdrawal latency assays. These findings provide evidence that L-CYSme can powerfully overcome the deleterious effects of morphine on breathing and gas-exchange in Sprague Dawley rats while not affecting the sedative or early stage antinociceptive effects of the opioid. The mechanisms by which L-CYSme interferes with the OR-induced signaling pathways that mediate the deleterious effects of morphine on ventilatory performance, and by which L-CYSme diminishes the late stage antinociceptive action of morphine remain to be determined.

## Introduction

The clinical usefulness of opioids analgesics, such as morphine and fentanyl, is compromised by their deleterious effects on breathing and alveolar gas exchange (van Dorp et al., 2007; Dahan et al., 2010; Dahan et al., 2018; Boom et al., 2012; Algera et al., 2019; Arendt, 2021). Opioid-induced respiratory depression (OIRD) can be overcome by administration of opioid receptor (OR) antagonists, such as naloxone, but these antagonists also block the analgesic and sedative actions of opioids, which may not be an issue in cases involving unexpected overdose, but which would be highly problematic in situations requiring analgesia/sedation, such as during and after surgery (Dahan et al., 2010; Dahan et al., 2018; Boom et al., 2012). Numerous classes of non-OR antagonist drugs have been proposed to combat OIRD (Dahan et al., 2010; Boom et al., 2012; van der Schier et al., 2014; Dahan et al., 2018; Algera et al., 2019; Imam et al., 2020). These drugs, many listed in Supplementary Table S1, include K + -channel blockers (Sia and Zandstra 1981), α2-adrenoceptor antagonists (Vonhof and Sirén, 1991), acetylcholinesterase inhibitors (Elmalem et al., 1991; Tsujita et al., 2007; Sakuraba et al., 2009), adenylate cyclase activators (Ballanyi et al., 1997), dopamine D1 receptor agonists (Ballanyi et al., 1997; Lalley, 2004; Lalley, 2005), phosphodiesterase inhibitors (Kasaba et al., 1997, Kimura et al., 2015), glycyl-glutamine (Owen et al., 2000), 5-HT_1A_-, 5-HT_1A_-/5-HT_7_- and 5-HT_4_-receptor agonists (Sahibzada et al., 2000; Manzke et al., 2003; Meyer et al., 2006; Dutschmann et al., 2009; Guenther et al., 2009; Manzke et al., 2011; Guenther et al., 2012; Ren et al., 2015), α-amino-3-hydroxy-5-methyl-4-isoxazole-propionic acid receptor (AMPA) agonists (ampakines) and brain targeted and allosteric AMPA receptor modulators (Ren et al., 2006; Greer and Ren, 2009; Ren et al., 2009; Lorier et al., 2010; Oertel et al., 2010; Cavalla et al., 2015; Haw et al., 2016; Dai et al., 2017; Sun et al., 2017; Dai et al., 2019; Xiao et al., 2020), the selective large conductance Ca^2+^-activated K^+^ (BK) channel blocker, GAL021 (Roozekrans et al., 2014; Golder et al., 2015; Roozekrans et al., 2015), which significantly improved alfentanil depression of ventilation in humans (Roozekrans et al., 2014; Roozekrans et al., 2015), the N-methyl-D-aspartate (NMDA) receptor antagonist, Esketamine (S-enantiomer of ketamine) (Jonkman et al., 2018), protein kinase A inhibitors, G protein-gated inwardly rectifying K^+^ (GIRK) channel blockers (Liang et al., 2018), microglial inhibitors (Hutchinson et al., 2008), thyrotropin releasing hormone and analogs (Boghosian et al., 2018), nicotinic receptor agonists (Ren et al., 2019, Ren et al., 2020), and KCNQ voltage-gated K^+^-channel blockers (Wei and Ramirez, 2019).

Most of the above OIRD reversal agents either did not enter into clinical trials, or did not progress satisfactorily in such trials (for reasons such as, lack of efficacy or unacceptable levels of side effects and/or toxicity), with a few others including ampakines and esketamine still under clinical evaluation (Dahan et al., 2018; Algera et al., 2019). As such, there is still an urgent unmet need to further develop drugs that effectively overcome OIRD by mechanisms other than direct blockade of ORs (Dahan et al., 2018; Algera et al., 2019). Recently, Algera et al. (2019) concluded that model-based drug development is needed in order to design an optimal reversal agent, which is not influenced by the kinetics of the interactions between opioid ligand and the OR, does not interfere with the analgesic actions of opioids, acts rapidly with sustained effects, and is devoid of as many adverse effects as possible. The conclusion based pharmacokinetic/pharmacodynamic data was that this ideal drug should act in the brainstem respiratory network via non-opioidergic pathways. These insights from Algera et al. (2019) are extremely valuable although issue could be taken as to whether the ideal drug should or should not directly combat OR-activated intracellular signaling processes that inhibit breathing, but not the OR-stimulated signaling pathways that elicit analgesia, and whether this ideal agent should act strictly in brainstem circuitry based on efficacy of peripherally restricted OR antagonists (Henderson et al., 2014). It is clear from Dahan et al. (2018), Algera et al. (2019) and Supplementary Table S1, that there is no unifying hypothesis as to the molecular mechanisms of action of putative ideal OIRD reversal agents.

There is compelling evidence that morphine inhibits the entry of L-cysteine into neurons via inhibition of the type 3 excitatory amino acid transporter (EEE3), resulting in subsequent changes in redox status (more oxidative environment) and/or the loss of L-cysteine participation in key signaling and metabolic pathways driving addiction (Trivedi et al., 2014; Trivedi and Deth, 2015). We hypothesize that this process may contribute to OIRD and reasoned that ethylester and methylester derivatives of cysteine and cysteine-containing compounds (i.e., thiolesters in which an ester linkage is attached to the carboxyl moiety) that can deliver the parent structure into cells may be a viable therapeutic strategy to overcome OIRD. As shown in Supplementary Table S2, there is an array of reduced (monothiol) and oxidized (disulfide) L-thiolesters that rapidly/readily enter cells within the peripheral and central nervous systems upon systemic administration or addition to *in vitro* preparations (references provided in Supplementary Table S2). Several L-thiolesters have been tested in disease/injury states, and for example, Henderson et al. (2016) demonstrated that γ-L-glutamyl cysteine ethylester (γ-L-GCee) decreased brain nitrosative stress in a rat traumatic brain injury model. The mechanisms by which L-thiolesters exert their biological effects are no doubt multi-factorial and may include (1) the formation of thiol adducts in the blood, such as D-glucose:L-cysteine (Wróbel et al., 1997; Szwergold, 2006; Li et al., 2015) and mixed disulfides (Wilcken and Gupta, 1979; Lash and Jones, 1985; Turell, et al., 2013), (2) direct docking of the parent L-thiolester with plasma membrane and/or intracellular proteins, such as ion-channels, receptors and enzymes that alters the activities of the proteins by mechanisms not associated with changes in redox status of the proteins (yet to be substantiated), (3) modulation of the redox status (e.g., cysteine-cystine switching) and function of plasma membrane proteins, such as Kv_1.2_ K^+^-channels (Baronas et al., 2017) and after entry into cells, redox modulation of functional intracellular proteins (Bogeski et al., 2011; Bogeski and Niemeyer, 2014; O-Uchi et al., 2014; Gamper and Ooi, 2015; Gao et al., 2017; García et al., 2018), (4) the formation of S-thiolated proteins, including S-cysteinylated, S-cysteinylglycinylated, S-glutathionylated and S-homocysteinylated proteins in plasma membranes and in cells (Winkler et al., 2007; Rossi et al., 2009; Auclair et al., 2013; Belcastro et al., 2017; Ghezzi and Chan, 2017; Bonifácio et al., 2021), (5) conversion of L-thiolesters to parent L-thiols by membrane associated esterases (Williams et al., 1985; Butterworth et al., 1993; Nishida et al., 1996; Satoh and Hosokawa, 1998; Satoh and Hosokawa, 2006; Hatfield et al., 2016) leading to increases in the intracellular levels of L-thiols, such as L-cysteine, which then enter into multiple metabolic pathways including those that generate hydrogen sulfide via the sequential actions of L-cysteine aminotransferase and cystathionine γ-lyase in central and peripheral tissues (Kimura, 2014; Kimura, 2017; Bełtowski, 2019) including the carotid bodies (Prabhakar, 2012), (6) conversion of L-thiolesters to cysteine sulfenics, sulfonics and sulfonics via cysteine dioxygenase (Yamaguchi and Hosokawa, 1987; Joseph and Maroney, 2007; Stipanuk et al., 2009; Stipanuk et al., 2011), and (7) formation of S-nitroso-L-cysteine, an endogenous S-nitrosothiol (Myers et al., 1990; Bates et al., 1991; Seckler et al., 2020) with many substantial roles in intracellular signaling cascades, (Lipton et al., 1993; Foster et al., 2009; Seth and Stamler, 2011; Stomberski et al., 2019; Gaston et al., 2020) including those controlling cardiorespiratory function (Davisson et al., 1996; Davisson et al., 1997; Ohta et al., 1997; Lipton et al., 2001; Gaston et al., 2006; Lewis et al., 2006; Gaston et al., 2020) and those involved in the attenuation of OIRD (Getsy et al., 2022a; Getsy et al., 2022b).

We are evaluating the abilities of monothiol and disulfide thiolesters to prevent and/or overcome OIRD in unanesthetized Sprague Dawley rats and reported that (1) pretreatment with L-glutathione ethylester (L-GSHee) diminished the adverse effects of fentanyl on ventilation and Alveolar-arterial (A-a) gradient (index gas alveolar exchange), but did not diminish fentanyl-induced antinociception/sedation (Jenkins et al., 2021), and (2) D-cysteine ethylester (D-CYSee) (Getsy et al., 2022c; Getsy et al., 2022d) or D-cystine diethylester (D-CYSdiee), D-cystine dimethyl ester (D-CYSdime) (Gaston et al., 2021) overcome the adverse actions of morphine on breathing and arterial blood-gas (ABG) chemistry while minimally affecting morphine antinociception/sedation. It appears that unlike OR antagonists, such as naloxone, the L-, D-thiolesters may overcome the deleterious actions of opioids by mechanisms other than direct/allosteric blockade of ORs (Gaston et al., 2021; Jenkins et al., 2021; Getsy et al., 2022a; Getsy et al., 2022b). Intravenous injections of L-cysteine ethylester (L-CYSee), but not L-cysteine, elicit an immediate and sustained reversal of the adverse effects of morphine on ABG chemistry and A-a gradient in isoflurane-anesthetized rats with a tracheal tube inserted to bypass the upper airway (nasopharynx, tongue, larynx, vocal folds), but not in rats without a tracheal tube (Mendoza et al., 2013). This finding that L-cysteine was inactive suggests that the effects of L-CYSee were due to entry of the L-thiolester into cells that initiate intracellular signaling events that overcome OIRD, but also obstruct the upper airway (e.g., collapse of the larynx, closure of the vocal folds, flaccid tongue that retracts and occludes the airway). The adverse effects of a combination of L-CYSee and morphine on the upper airway may involve the pharmacological actions of isoflurane since these effects of L-CYSee were not seen in sevoflurane-anesthetized rat (Bates et al., 1991), which is the dominant clinical gaseous anesthetic (Aoki et al., 2021; Apai et al., 2021; Liang et al., 2021). Here we extend our knowledge of L-thiolesters by describing the actions of L-CYSme on the effects of morphine in unanesthetized, unrestrained male Sprague Dawley rats. Similar to L-CYSee, L-CYSme has been widely used in studies designed to understand the effects of thiolesters on redox and metabolic processes (Supplementary Table S2). We report here that intravenous injections of L-CYSme (500 μmol/kg), but not L-cysteine (500 μmol/kg), elicit a rapid and sustained reversal of the adverse effects of 10 mg/kg of morphine on ventilatory parameters, A-a gradient and ABG chemistry while not affecting opioid antinociception/sedation. However, a distinct decrease in duration, but not maximal level of morphine-induced antinociception or sedation occurred with 5 mg/kg morphine. This pharmacological profile of L-CYSme would be advantageous in scenarios requiring immediate reversal of the deleterious effects of opioids on breathing and especially when opioids are essential for pain relief thereby making administration of popular OR antagonists a problematic situation.

## Materials and methods

### Permissions, rats, and surgical procedures

All studies were carried out in strict accordance with the NIH Guide for Care and Use of Laboratory Animals (NIH Publication No. 80–23) revised in 1996, and in strict compliance with ARRIVE (Animal Research: Reporting of *In Vivo* Experiments) guidelines (http://www.nc3rs.org.uk/ page. asp? id = 1,357). All protocols involving the use of rats were approved by the Animal Care and Use Committees of Galleon Pharmaceuticals, Case Western Reserve University, and the University of Virginia. Adult male Sprague Dawley rats were purchased from *Harlan Industries* (Madison, WI, United States). After 5 days of recovery from transportation, the rats received femoral artery catheters and/or jugular vein catheters under 2–3% isoflurane anesthesia (Henderson et al., 2014; Gaston et al., 2021). The rats were given 4 days to recover from surgery before use. All femoral arterial catheters were flushed daily with a heparin solution (50 units heparin in 0.1 M, pH 7.4 phosphate-buffered saline). On the day of the study, all arterial and venous catheters were flushed with 0.3 ml of phosphate-buffered saline (0.1 M, pH 7.4) 3–4 h before commencement of the study. The pH of all stock solutions of vehicle, L-cysteine and L-CYSme were adjusted to pH of 7.2 with 0.25 M NaOH. All studies were done in a quiet room with relative humidity of 50 ± 2% and room temperature of 21.3 ± 0.2 °C. The antinociception and ventilatory/ABG chemistry experiments were performed in separate groups of rats to not compromise the ventilatory measurements. The plethysmography and antinociception recording sessions and the arterial blood sampling studies (ABG assays) were done by an investigator who injected the opioid, vehicle, L-cysteine or L-CYSme. The syringes with vehicle or test drug were made up by another investigator, such that the person performing the injections was blinded to the treatment protocol. A liquid form of (+)-morphine sulfate (10 mg/ml) was obtained from Baxter Healthcare Corporation (Deerfield, IL, United States). L-CYSme HCl powder (Product number: 410209; PubChem Substance ID: 24865699) was obtained from Sigma-Aldrich (St. Louis, MO, United States) and divided into 100 mg amounts under N_2_ gas and stored at 4°C. Solutions of L-CYSme (dissolved in saline and brought to pH 7.2 with 0.1 M NaOH at room temperature) were prepared immediately before injection. In every case, the data files resulting from each study were collated and analyzed by another investigator in the group blinded to the treatment protocol.

### Protocols for whole body plethysmography measurement of ventilatory parameters

Ventilatory parameters were recorded continuously in the unrestrained freely-moving rats using a whole body plethysmography system (PLY3223; Data Sciences International, St. Paul, MN) as detailed previously May et al., 2013a; May et al., 2013b; Young et al., 2013; Getsy et al., 2014; Henderson et al., 2014; Baby et al., 2018; Gaston et al., 2020; Baby S. et al., 2021; Baby S. M. et al., 2021; Getsy et al., 2021; Gaston et al., 2021; Seckler et al., 2022). The directly recorded and calculated (derived) parameters are defined in Supplementary Table S3 (Hamelmann et al., 1997; Lomask, 2006; Tsumuro et al., 2006; Quindry et al., 2016; Gaston et al., 2021). The directly recorded and derived ventilatory parameters, and the abbreviations used in this manuscript are as follows: frequency of breathing (Freq), tidal volume (TV), minute ventilation (MV), inspiratory time (Ti), expiratory time (Te), Ti/Te, end inspiratory pause (EIP), end expiratory pause (EEP), peak inspiratory flow (PIF), peak expiratory flow (PEF), PIF/PEF ratio, expiratory flow at 50% expired TV (EF_50_), relaxation time (RT), inspiratory drive (TV/Ti), expiratory drive (TV/Te), apneic pause [(Te/RT)-1], non-eupneic breathing index (NEBI) and NEBI corrected for Freq (NEBI/Freq). A diagram of the relationships between a few of the directly recorded parameters (adapted from Lomask, 2006) are shown in Supplementary Figure S1. On the day of the study, each rat was placed in an individual plethysmography chamber and allowed at least 60 min to acclimatize so that resting (i.e., baseline or, pre) ventilatory parameter values were accurately defined. Two sets of studies were performed. Study 1: Two groups of rats received a bolus injection of morphine (10 mg/kg, IV) and after 15 min, one group received a bolus injection of vehicle (saline) and the other group received a bolus injection of L-CYSme (500 μmol/kg, IV). The rats then received a second injection of vehicle or L-CYSme (500 μmol/kg, IV) 15 min later. Study 2: Two groups of rats received a bolus injection of morphine (10 mg/kg, IV) and after 15 min, one group received an injection of vehicle (saline) and the other group received an injection of L-cysteine (500 μmol/kg, IV). The rats then received a second injection of vehicle or L-cysteine (500 μmol/kg, IV) 15 min later. Ventilatory parameters were monitored for 60 min after the second injection of vehicle, L-cysteine or L-CYSme. The body weights of all groups of rats were similar to one another and as such, ventilatory parameters related to volumes (e.g., TV, PIF, PEF, EF_50_) are presented without correcting for body weight. The FinePointe (DSI) software constantly corrected digitized ventilatory values originating from the actual waveforms for alterations in chamber humidity and temperature. Pressure changes associated with the respiratory waveforms were then converted to volumes (e.g., TV, PIF, PEF, EF_50_) employing the algorithms of Epstein and colleagues (Epstein and Epstein, 1978; Epstein et al., 1980). Specifically, factoring in chamber humidity and temperature, cycle analyzers filtered the acquired signals, and FinePointe algorithms generated an array of box flow data that identified a waveform segment as an acceptable breath. From that data vector, the minimum and maximum values were determined and multiplied by a compensation factor provided by the selected algorithm (Epstein and Epstein, 1978; Epstein et al., 1980) thus producing TV, PIF and PEF values that were used to determine non-eupneic breathing events expressed as the non-eupneic breathing index (NEBI), reported as the percentage of non-eupneic breathing events per epoch (Getsy et al., 2014). Apneic pause was determined by the formula, (Expiratory Time/Relaxation Time)—1 (Gaston et al., 2021).

### Protocols for blood gas measurements and determination of arterial-alveolar gradient

Changes in ABG chemistry values (pH, pCO_2_, pO_2_ and sO_2_) and A-a gradients elicited by an injection of morphine (10 mg/kg, IV) in three different sets of unanesthetized freely-moving rats (n = 9 rats per group) and after an injection of vehicle (saline, IV; 82.2 ± 0.4 days of age; 335 ± 2 g body weight), L-cysteine (500 μmol/kg, IV; 82.6 ± 0.5 days; 336 ± 3 g) or L-CYSme (500 μmol/kg, IV; 81.7 ± 0.5 days; 332 ± 2 g) were determined as detailed previously (Henderson et al., 2014; Baby et al., 2018; Baby S. et al., 2021; Gaston et al., 2021). Briefly, samples of arterial blood (100 μl) were taken 15 min before and 15 min after injection of morphine (10 mg/kg, IV). The rats then immediately received an injection of vehicle, L-cysteine or L-CYSme and blood samples were taken at 5, 15, 30 and 45 min time points. The pH, pCO_2_, pO_2_ and sO_2_ were determined by a Radiometer blood-gas analyzer (ABL800 FLEX). The A-a gradient defines differences between alveolar and arterial blood O_2_ concentrations (Stein et al., 1995; Story 1996; Henderson et al., 2014). A reduction in PaO_2_, without a concomitant alteration in A-a gradient is the result of hypo-ventilation, whereas a decrease in PaO_2_ with a concomitant elevation in A-a gradient indicates an on-going mismatch in ventilation-perfusion in alveoli (Stein et al., 1995; Story 1996; Henderson et al., 2014; Gaston et al., 2021). A-a gradient = PAO_2_ − PaO_2_, where PAO_2_ is the partial pressure (p) of alveolar O_2_ and PaO_2_ is pO_2_ in the sampled arterial blood. PAO_2_ = [(FiO_2_ x (P_atm_ - P_H2O_) - (PaCO_2_/respiratory   quotient)], where FiO_2_ is the fraction of O_2_ in inspired air; P_atm_ is atmospheric pressure; P_H2O_ is the partial pressure of H_2_O in inspired air; PaCO_2_ is pCO_2_ in arterial blood; and respiratory quotient (RQ) is the ratio of CO_2_ eliminated/O_2_ consumed. We took FiO_2_ of room-air to be 21% = 0.21, P_atm_ to be 760 mmHg, and P_H2O_ to be 47 mmHg (Gaston et al., 2021). We took the RQ value of our adult male rats to be 0.9 (Stengel et al., 2010; Chapman et al., 2012; Gaston et al., 2021; Jenkins et al., 2021).

### Antinociception assessment by tail-flick latency assay

The antinociceptive effects elicited by an injection of morphine and a subsequent injection of vehicle or D-CYSme were determined by evaluating tail-flick latency (TFL) with the use of a Tail-Flick Analgesia Meter (IITC Life Science Inc., United States) as described previously (Lewis et al., 1991; Meller et al., 1991; Carstens and Wilson, 1993; Henderson et al., 2014; Golder et al., 2015; Gaston et al., 2021; Jenkins et al., 2021). This procedure entailed a minor level of manual restraint during the positioning of the tail to apply a thermal stimulus sufficient to induce a latency of tail withdrawal of about 3.0 s in all rats. In reversal studies, baseline TFL was tested in all rats prior to any drug administration (-20 min time point). One group of rats (81.2 ± 0.9 days of age; 337 ± 2 g body weight, *n* = 6) received an injection of morphine (5 mg/kg, IV) and after TFL testing at + 20 min they immediately received a bolus IV injection of vehicle (saline, 100 μL/100 g body weight). A second group of rats (80.5 ± 1.5 days; 336 ± 3 g, *n* = 4) received a bolus injection of L-CYSme (500 μmol/kg, IV) at the +20 min time point. A third group of rats (80.7 ± 1.1 days of age; 335 ± 2 g body weight, *n* = 6) received an injection of morphine (10 mg/kg, IV) and after TFL testing at + 20 min they immediately received a bolus IV injection of vehicle (saline, 100 μL/100 g body weight). A fourth group of rats that had received 10 mg/kg morphine, IV (81.5 ± 1.6 days; 336 ± 3 g, *n* = 6) received a bolus injection of L-CYSme (500 μmol/kg, IV) at the +20 min time point. TFL was tested 20, 40, 60 , 70, 100 and 160 min post-injection of vehicle or L-CYSme. The resulting data are presented as actual TFL (sec) and as maximum possible effect (%MPE) determined by the formula, %MPE = [(post-injection TFL − baseline TFL)/(12 − baseline TFL)] x 100 (Lewis et al., 1991; Meller et al., 1991; Carstens and Wilson, 1993; Henderson et al., 2014; Golder et al., 2015; Gaston et al., 2021; Jenkins et al., 2021). In pre-treatment studies, baseline TFL was tested in all rats prior to any drug administration (−20 min time point). One group of rats (81.2 ± 0.5 days of age; 330 ± 2 g body weight, n = 9) then received a bolus IV injection of vehicle (saline, 100 μL/100 g body weight). A second group (82.0 ± 0.4 days; 334 ± 2 g, n = 9) received a bolus injection of L-CYSme (500 μmol/kg, IV). A third group (79.8 ± 0.4 days; 329 ± 3 g, *n* = 6) received a bolus injection of L-cysteine (500 μmol/kg, IV). TFL was then tested in the three groups 10 and 20 min after these injections (i.e., at the -10 and 0 min time points, respectively). At +20 min post-injection (time point 0 min), the rats got an injection of morphine (10 mg/kg, IV) and TFL was tested 20 , 40 , 60 , 90 , 120, 150, 180, 210, 240, 360 and 480 min post-injection. In another study, one group of rats (82.2 ± 0.4 days of age; 336 ± 3 g, *n* = 9) received an injection of vehicle (saline, 100 μL/100 g body weight) and a second group (82.5 ± 0.4 days; 339 ± 2 g, *n* = 9) received an injection of L-CYSme (500 μmol/kg, IV) and then an IV injection of a 5 mg/kg dose of morphine and TFL was tested as above. TFL data are presented as actual TFL (sec) and as maximum possible effect (%MPE) determined by the formula, %MPE = [(post-injection TFL − baseline TFL)/(12 − baseline TFL)] x 100 (Lewis et al., 1991; Meller et al., 1991; Carstens and Wilson, 1993; Henderson et al., 2014; Golder et al., 2015; Gaston et al., 2021; Jenkins et al., 2021).

### Antinociception assessment by paw withdrawal assay

The antinociceptive actions of morphine, vehicle, L-cysteine and L-CYSme were assessed by hot-plate (hind-paw withdrawal) latency (HPL) assay using the Hargreaves’s method (Hargreaves et al., 1988), as detailed previously (Baby S. et al., 2021; Gaston et al., 2021). In brief, HPL in response to a thermal stimulus was assessed using a radiant heat source (IITC, CA, United States) aimed at the plantar surface of the left hind-paw. This technique did not require restraint of the rat while positioning the thermal stimulus that was sufficient to produce paw withdrawal from the floor of the hot-plate in about 20 s (baseline values) before injection of a drug (cut-off latency was 40 s to avoid any tissue damage). Baseline HPL was tested in all three groups (−20 min time-point). One group of rats (79.7 ± 0.3 days of age; 334 ± 3 g body weight, *n* = 9) received an IV injection of vehicle (saline, 100 μl/100 g body weight). A second group (80.0 ± 0.3 days; 337 ± 3 g, *n* = 9) received an injection of L-cysteine (500 μmol/kg, IV). A third group (80.1 ± 0.3 days; 336 ± 3 g, *n* = 9) received an injection of L-CYSme (500 μmol/kg, IV). HPL was tested 10 and 20 min later (i.e., at the -10 and 0 min time points). At +20 min post-injection (time point 0 min), all rats received an injection of morphine (10 mg/kg, IV) and HPL was tested 15, 30, 60, 90 , 120, 180, 210, 240, 360 and 480 min post-injection. The data are presented as HPL (sec) and as “maximum possible effect” (%MPE) determined by the formula, %MPE = [(post-injection HPL − baseline HPL)/(20 − baseline HPL)] x 100.

### Sedation as determined by the modified righting reflex test

This study evaluated the effects of bolus injections of vehicle, L-cysteine (500 μmol/kg, IV) and L-CYSme (500 μmol/kg, IV) on the duration of morphine (10 mg/kg, IV) on the modified righting reflex test. Each rat was placed in an open plastic chamber to evaluate the duration of loss of the modified righting reflex. The injection of morphine caused the rats to assume numerous types of postures, including being motionless sprawled out on their stomach on the chamber floor, lying motionless on their side, and splayed out on their stomach with the head up against the chamber wall. The duration of the sedative effects of morphine was taken as the time interval from the time of morphine injection to full recovery of the righting reflex (i.e., when rats attained/maintained a normal posture on all four legs (Ren et al., 2015; Yu et al., 2018; Ren et al., 2020; Jenkins et al., 2021). One group of rats (79.6 ± 0.3 days; 328 ± 2 g, *n* = 9) received an injection of morphine (10 mg/kg, IV) and after 15 min an injection of vehicle (saline). A second group (79.5 ± 0.3 days; 331 ± 3 g, *n* = 9) received an injection of morphine (10 mg/kg, IV) and after 15 min an injection of L-cysteine (500 μmol/kg, IV). A third group (79.4 ± 0.3 days; 327 ± 3 g, *n* = 9) received a bolus injection of morphine (10 mg/kg, IV) and after 15 min an injection of L-CYSme (500 μmol/kg, IV).

### Data analyses

The directly recorded and arithmetically-derived parameters (1 min bins) were taken for statistical analyses. Pre-drug 1 min bins excluded occasional marked deviations from resting values due to abrupt movements by the rats, such as scratching. The exclusions ensured accurate determination of baseline parameters. All data are presented as mean ± SEM and were evaluated using one-way and two-way ANOVA followed by Bonferroni corrections for multiple comparisons between means using the error mean square terms from each ANOVA analysis (Wallenstein et al., 1980; Ludbrook, 1998; McHugh, 2011) as detailed previously (Getsy et al., 2021). A *p* < 0.05 value denoted the initial level of statistical significance that was modified according to the number of comparisons between means as described by Wallenstein et al. (1980). The modified *t-*statistic is t = (mean group 1 - mean group 2)/[s x (1/n_1_ + 1/n_2_)^1/2^] where s^2^ = the mean square within groups term from the ANOVA (the square root of this value is used in the modified *t*-statistic formula) and n_1_ and n_2_ are the number of rats in each group under comparison. Based on an elementary inequality called Bonferroni’s inequality, a conservative critical value for modified *t*-statistics obtained from tables of *t*-distribution using a significance level of P/m, where m is the number of comparisons between groups to be performed (Winer, 1971). The degrees of freedom are those for the mean square for within group variation from the ANOVA table. In the majority of situations, the critical Bonferroni value cannot be found in conventional tables of the t- distribution but can be approximated from tables of the normal curve by t* = z + (z + z^3^)/4n, with n being the degrees of freedom and z being the critical normal curve value for P/m (Wallenstein et al., 1980; Ludbrook, 1998; McHugh, 2011). Wallenstein et al. (1980) first demonstrated that the Bonferroni procedure is preferable for general use since it is easy to apply, has the widest range of applications, and because it provides critical values that are lower than those of other procedures when the investigator can limit the number of comparisons (and will be slightly larger than those of other procedures if many comparisons are made. As mentioned, a value of *p* < 0.05 was taken as the initial level of statistical significance (Wallenstein et al., 1980; Ludbrook, 1998; McHugh, 2011 and statistical analyses were performed with the aid of GraphPad Prism software (GraphPad Software, Inc., La Jolla, CA).

## Results

### Effects of L-cysteine or L-CYSme on the ventilatory responses to morphine

**FIGURE 1:**
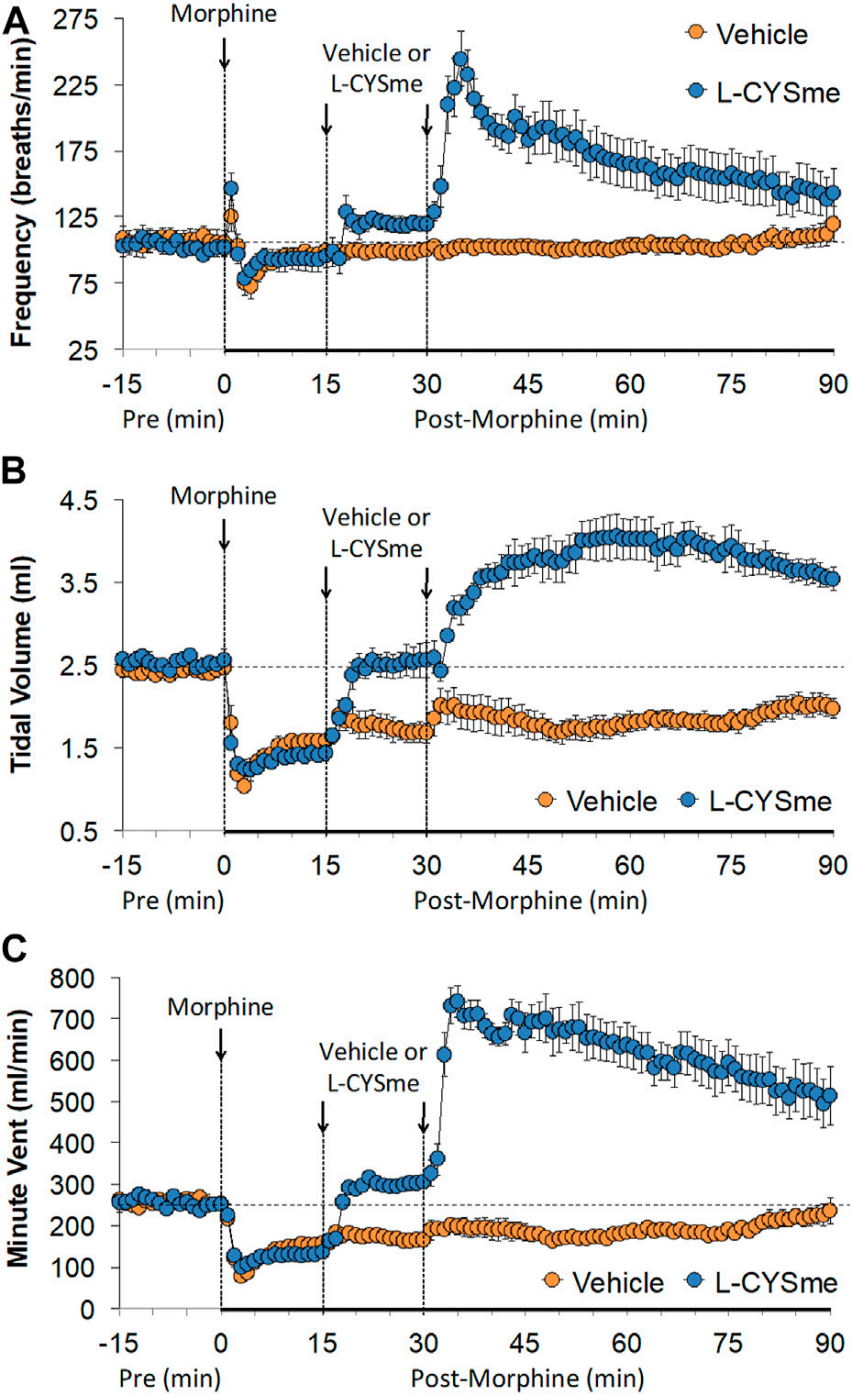
Frequency of breathing Panel **(A)**, tidal volume Panel **(B)** and minute ventilation Panel **(C)** prior to (Pre), following the injection of morphine (10 mg/kg, IV), and then two injections of vehicle or L-cysteine methyl ester (L-CYSme, 500 μmol/kg, IV) given 15 min apart, in freely-moving rats. The data are presented as mean ± SEM. There were 6 rats in each group.

The ages, body weights, and baseline (pre) ventilatory parameter values of the rats before the administration of morphine (10 mg/kg, IV) are shown in Supplementary Table S4. There were no between group differences for any of the parameters (*p* > 0.05, for all comparisons). A summary of the total responses produced by the injection of morphine (10 mg/kg, IV) during the 15 min prior to the rats receiving injections of vehicle or L-CYSme are described in Supplementary Table S5. The injection of morphine elicited pronounced decreases in TV, MV, PIF, PIF/PEF and inspiratory and expiratory drives that were accompanied by substantial increases in Ti, Te, Ti/Te, EIP, EEP, apneic pause, NEBI and NEBI/Freq. There were no sustained changes in Freq, Te, PEF, EF_50_ or RT. Figure 1 summarizes the minute-by-minute values for Freq, TV and MV recorded before, after the injection of morphine (10 mg/kg, IV) and subsequent injections of vehicle or L-CYSme (500 μmol/kg, IV). As seen in Panel A, the injection of morphine elicited a transient increase in Freq that was followed by a relatively minor decrease in Freq in both groups that had recovered towards baseline values at the time (15 min post-injection of morphine) that vehicle or L-CYSme was injected. The two injections of vehicle given 15 min apart elicited negligible responses (Panel A). Panel A also shows that the first injection of L-CYSme elicited a relatively small but sustained increase in Freq, whereas the second injection elicited a pronounced elevation in Freq that gradually declined towards resting values over the following 60 min. As can be seen in Panel B, the injection of morphine elicited a rapid, substantial, and sustained decrease in TV in both groups of rats and this decrease in TV remained 15 min post-administration of morphine when the injections of vehicle or L-CYSme were given The injections of vehicle did not immediately change TV, which gradually recovered to pre-morphine levels toward the end of the recording period (90 min post-morphine). As also seen in Panel B, the first injection of L-CYSme elicited a prompt and sustained reversal of the deleterious effects of morphine on TV (to values equivalent to those prior to administration of morphine), whereas the second injection elicited a remarkable elevation in TV that had not fully recovered to baseline values after 60 min. As can be seen in Panel C, the above changes in Freq and TV translated into sustained morphine-induced decreases in MV and a prompt and sustained reversal by L-CYSme of the deleterious effects of morphine on MV.

**FIGURE 2:**
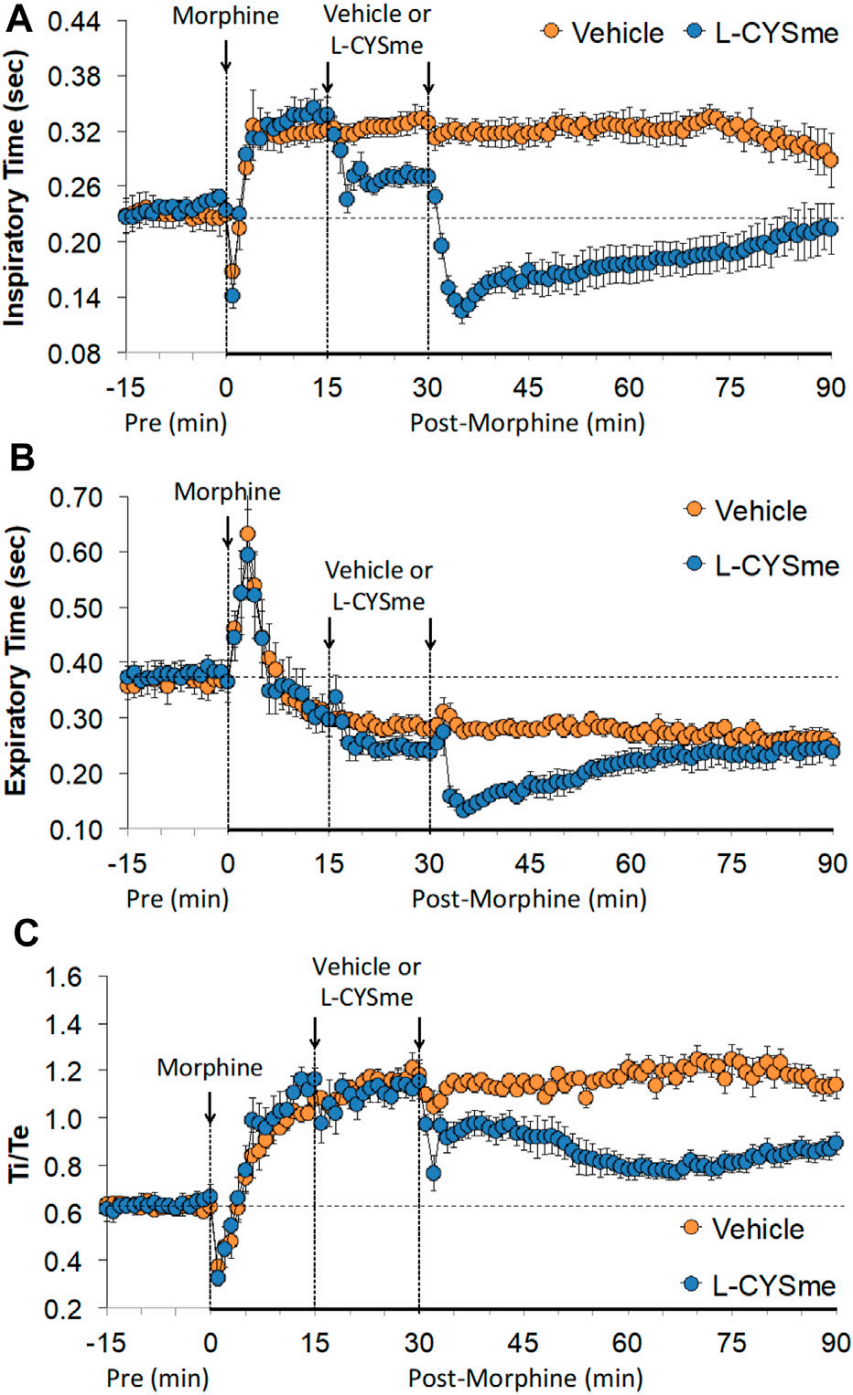
Inspiratory time Panel **(A)**, expiratory time Panel **(B**) and inspiratory time/expiratory time (Ti/Te) Panel **(C)** prior to (Pre), following the injection of morphine (10 mg/kg, IV), and then two injections of vehicle or L-cysteine methyl ester (L-CYSme, 500 μmol/kg, IV) given 15 min apart, in freely-moving rats. The data are shown as mean ± SEM. There were 6 rats in each group.

**FIGURE 3:**
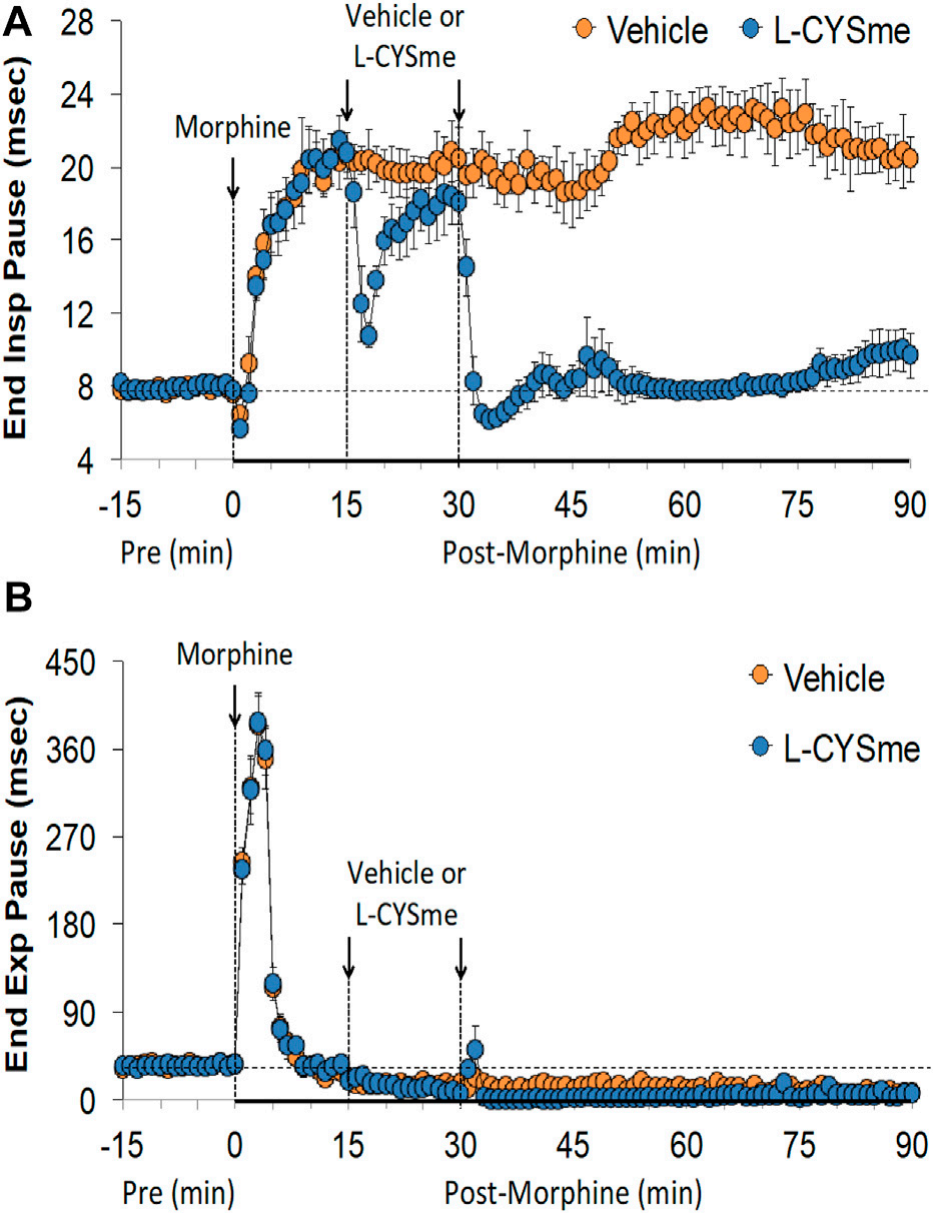
End inspiratory pause Panel **(A)** and end expiratory pause Panel **(B)** prior to (Pre), following the injection of morphine (10 mg/kg, IV), and then two injections of vehicle or L-cysteine methyl ester (L-CYSme, 500 μmol/kg, IV) given 15 min apart, in freely-moving rats. The data are presented as mean ± SEM. There were 6 rats in each group.

**FIGURE 4:**
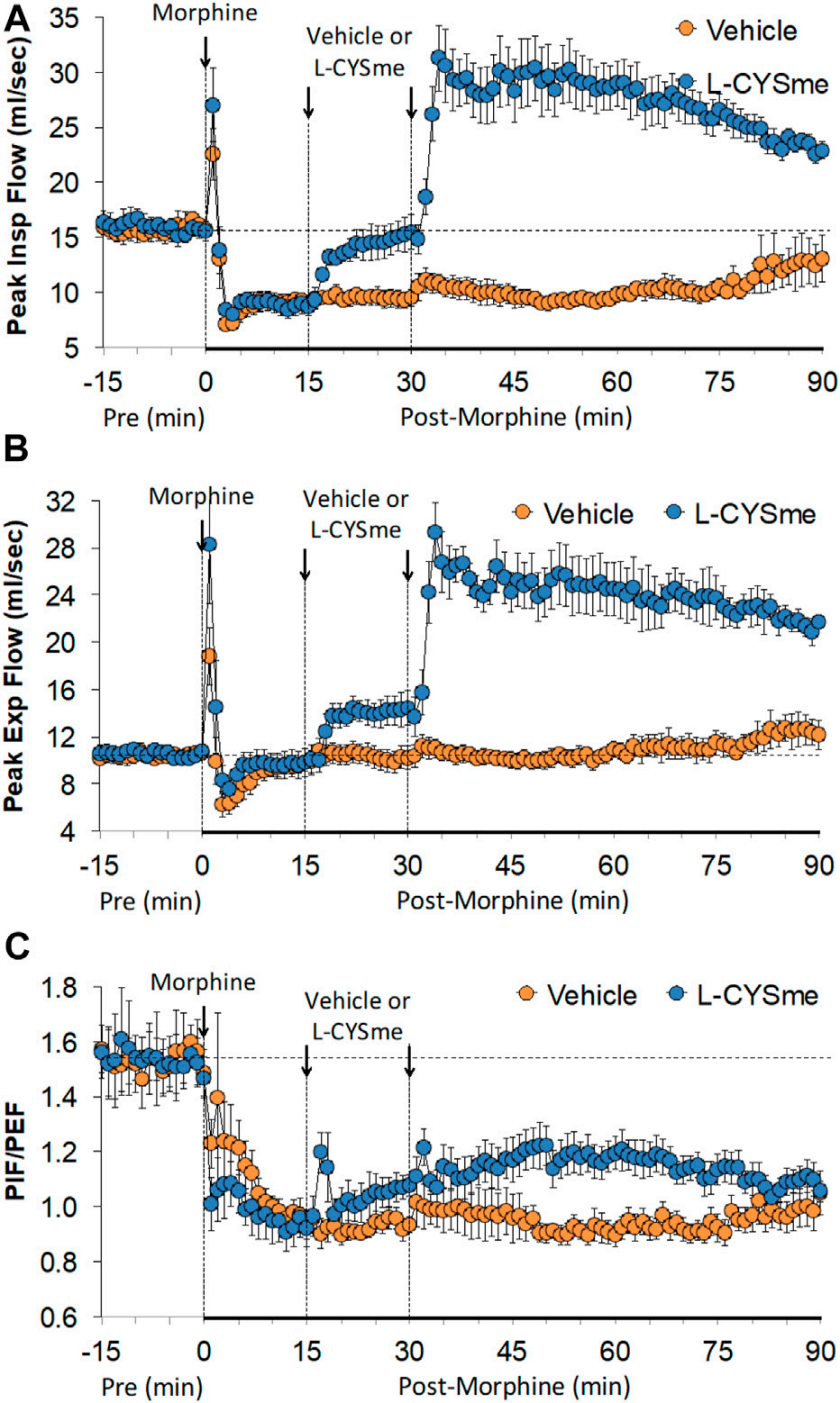
Peak inspiratory flow (Panel **(A)**, peak expiratory flow (Panel **(B)** and peak inspiratory flow/peak expiratory flow (PIF/PEF) (Panel **(C)** prior to (Pre), following the injection of morphine (10 mg/kg, IV), and then two injections of vehicle or L-cysteine methyl ester (L-CYSme, 500 μmol/kg, IV) given 15 min apart, in freely-moving rats. The data are presented as mean ± SEM. There were 6 rats in each group.

**FIGURE 5:**
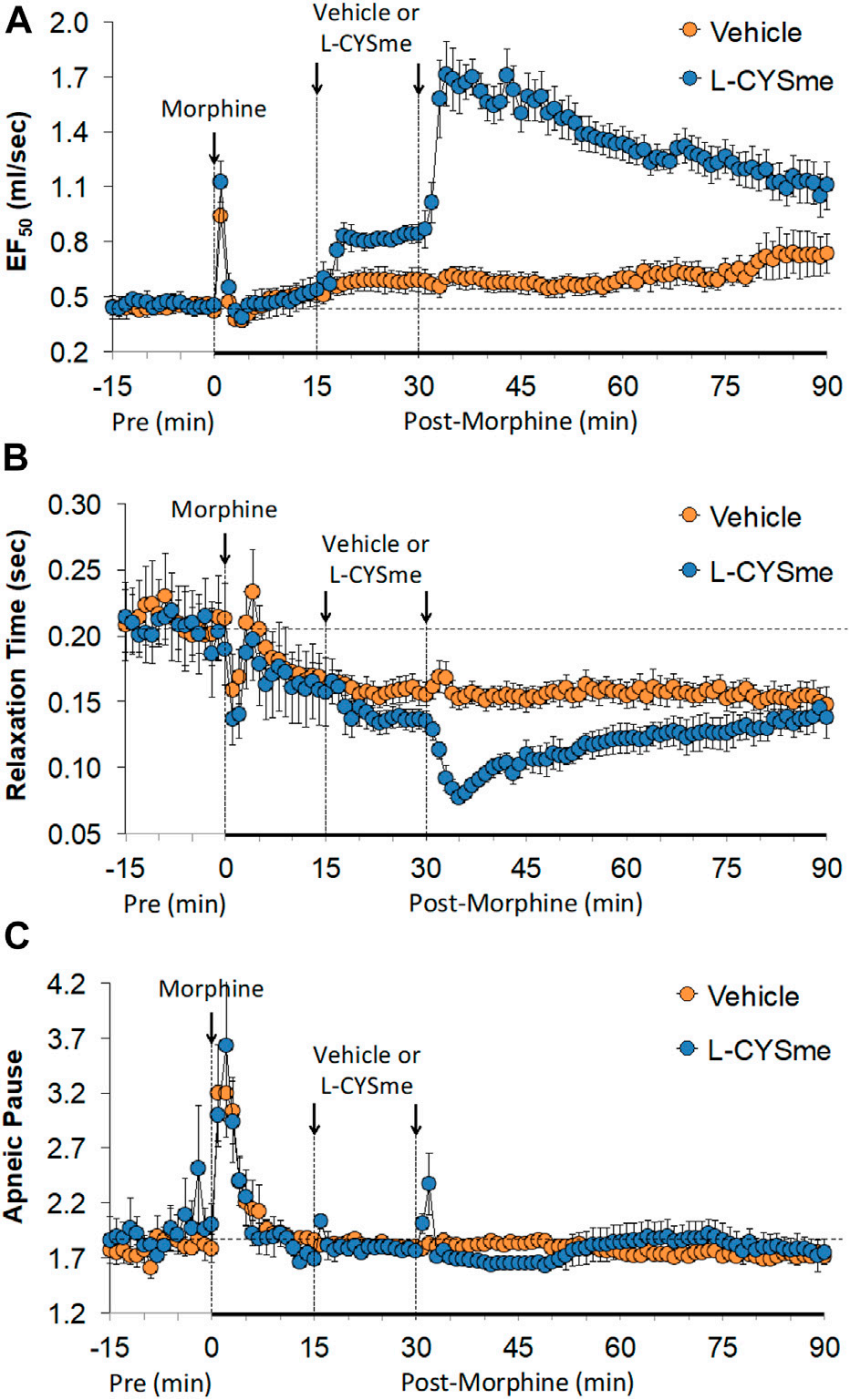
Expiratory flow at 50% of expired tidal volume (EF_50_) Panel **(A)**, relaxation time Panel **(B)** and apneic pause Panel **(C)** prior to (Pre), following the injection of morphine (10 mg/kg, IV), and then two injections of vehicle or L-cysteine methyl ester (L-CYSme, 500 μmol/kg, IV) given 15 min apart, in freely-moving rats. The data are presented as mean ± SEM. There were 6 rats in each group.

**FIGURE 6:**
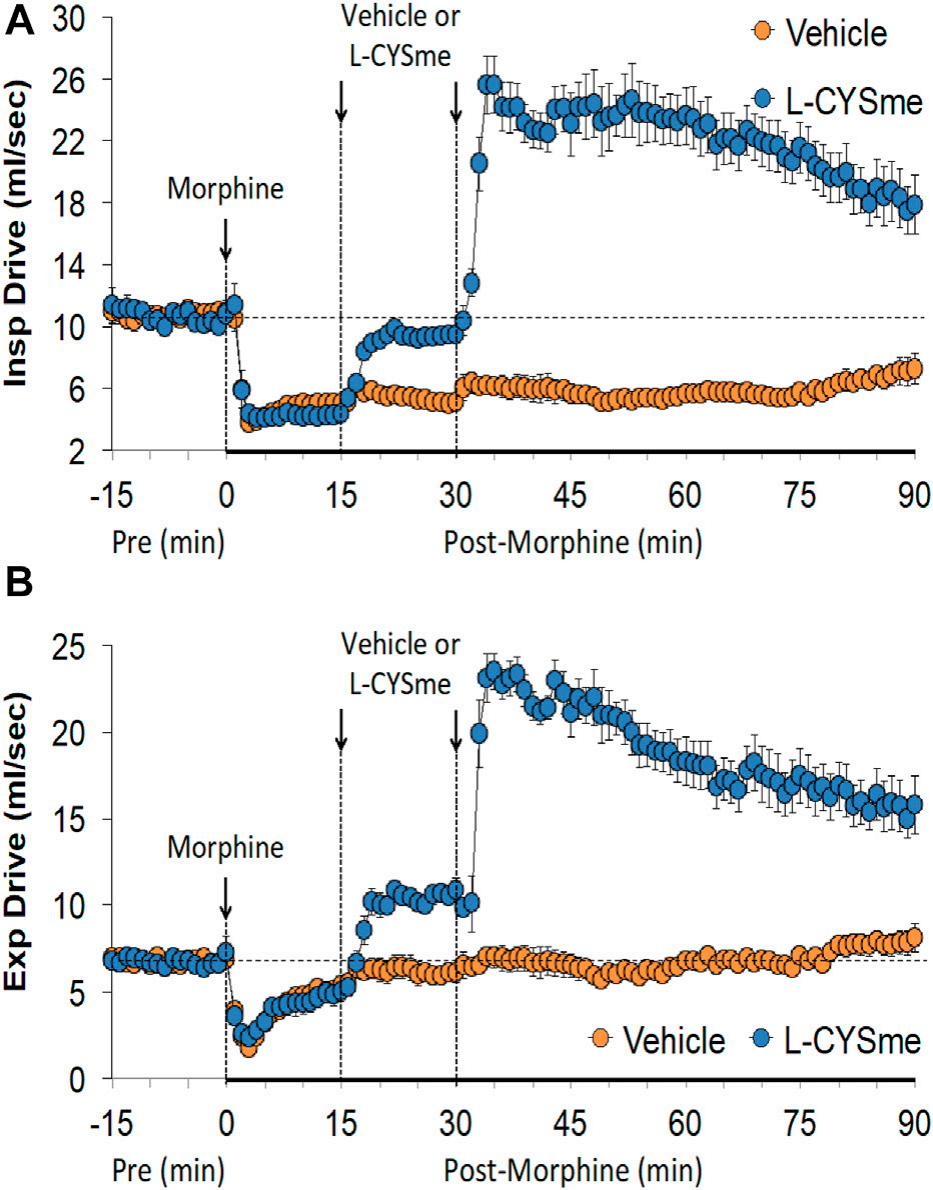
Inspiratory drive (Panel **(A)** and expiratory drive (Panel **(B)** prior to (Pre), following the injection of morphine (10 mg/kg, IV), and then two injections of vehicle or L-cysteine methyl ester (L-CYSme, 500 μmol/kg, IV) given 15 min apart, in freely-moving rats. The data are presented as mean ± SEM. There were 6 rats in each group.

**FIGURE 7:**
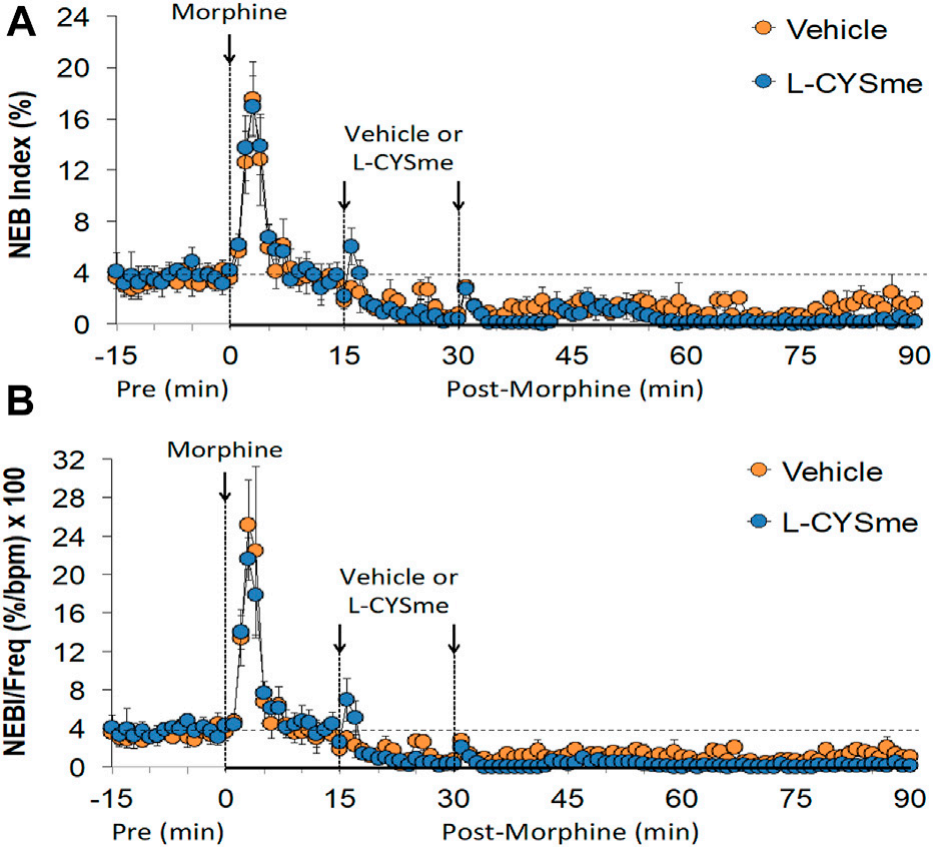
Non-eupneic breathing (NEB) index Panel **(A)** and NEB index/frequency of breathing (Panel **(B)** prior to (Pre), following the injection of morphine (10 mg/kg, IV), and then two injections of vehicle or L-cysteine methyl ester (L-CYSme, 500 μmol/kg, IV) given 15 min apart, in freely-moving rats. The data are presented as mean ± SEM. There were 6 rats in each group.

**FIGURE 8:**
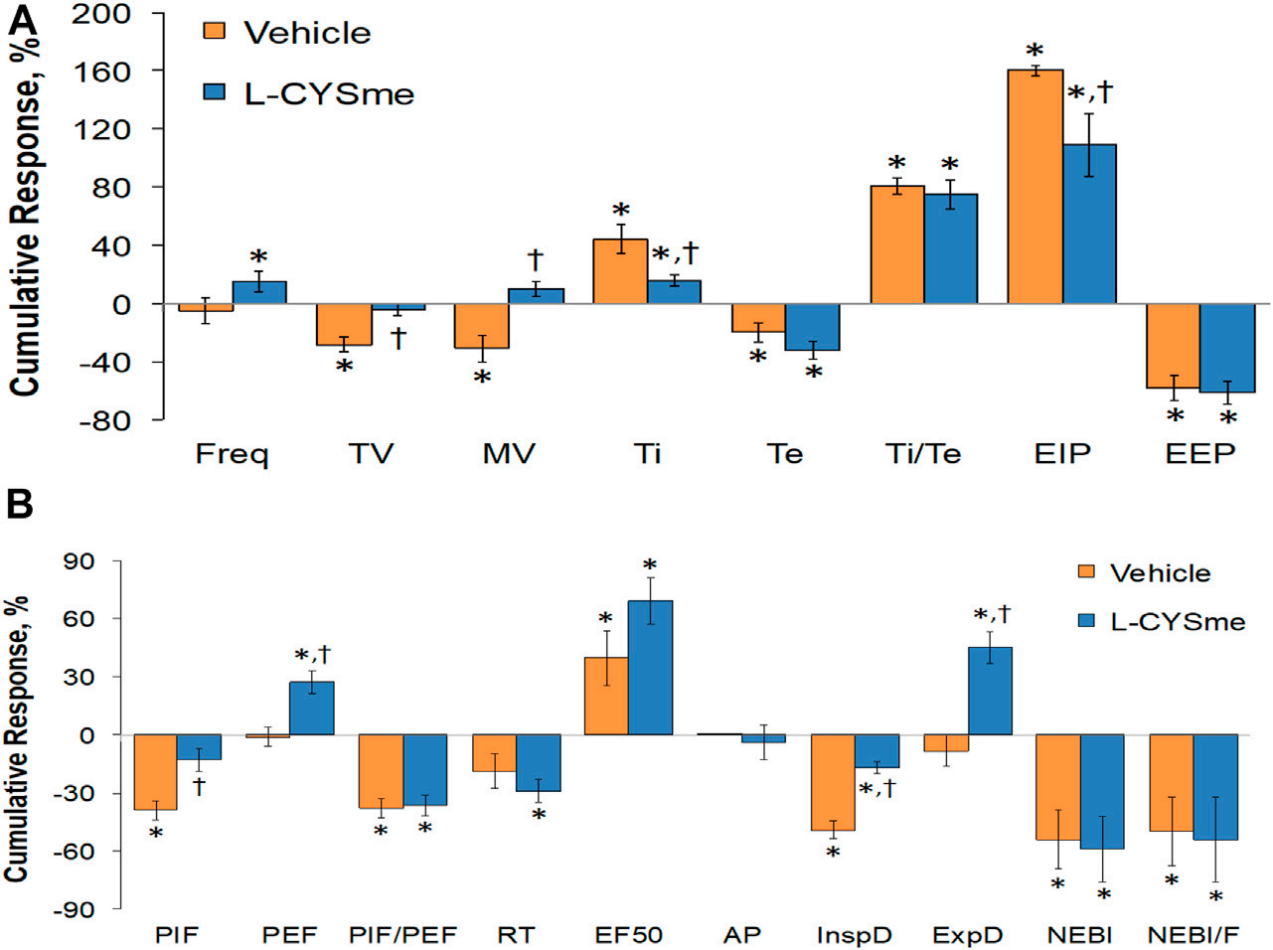
Cumulative changes in ventilatory parameters recorded over the 15-min period following the first injection of vehicle or L-cysteine methyl ester (L-CYSme, 500 μmol/kg, IV) in rats that had been injected with morphine (10 mg/kg, IV). Panel **(A)** frequency of breathing (Freq), tidal volume (TV), minute ventilation (MV), inspiratory time (Ti), expiratory time (Te), Ti/Te, end inspiratory pause (EIP), and end expiratory pause (EEP). Panel **(B)** peak inspiratory flow (PIF), peak expiratory flow (PEF), PIF/PEF, relaxation time (RT), expiratory flow at 50% of expired tidal volume (EF_50_), apneic pause (AP), inspiratory drive (InspD, TV/Ti), expiratory drive (ExpD, TV/Te), non-eupneic breathing index (NEBI), and NEBI/freq (NEBI/F). Data are presented as mean ± SEM. There were 6 rats in each group. *p < 0.05, significant difference from Pre values. ^†^ *p* < 0.05, L-CYSme *versus* vehicle.

**FIGURE 9:**
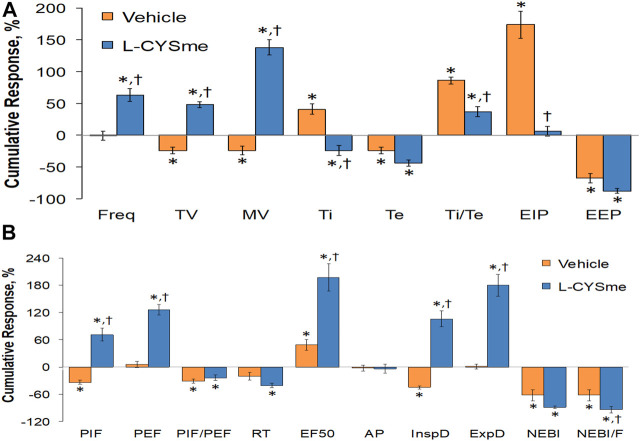
Cumulative changes in ventilatory parameters recorded over the 60-min period that followed the second injection of vehicle or L-cysteine methyl ester (L-CYSme, 500 μmol/kg, IV) in rats injected with morphine (10 mg/kg, IV). Panel **(A)** frequency of breathing (Freq), tidal volume (TV), minute ventilation (MV), inspiratory time (Ti), expiratory time (Te), Ti/Te, end inspiratory pause (EIP), and end expiratory pause (EEP). Panel **(B)** peak inspiratory flow (PIF), peak expiratory flow (PEF), PIF/PEF, relaxation time (RT), expiratory flow at 50% of expired tidal volume (EF_50_), apneic pause (AP), inspiratory drive (InspD, TV/Ti), expiratory drive (ExpD, TV/Te), non-eupneic breathing index (NEBI), and NEBI/freq (NEBI/F). Data are presented as mean ± SEM. There were 6 rats in each group. *p < 0.05 significant difference from Pre values. ^†^ *p* < 0.05, significant difference between L-CYSme *versus* vehicle.

As shown in Figure 2 (Panel A), the injection morphine elicited a transient decrease in Ti that was followed by sustained increases in Ti in both groups of rats. The two injections of vehicle elicited minor changes from the increase in Ti seen after morphine injection, whereas the first and especially the second injection of L-CYSme elicited substantial decreases in Ti. As seen in Panel B, morphine elicited a transient increase in Te that was followed by a sustained decrease in Te. The subsequent injections of vehicle did not produce noticeable responses, whereas the second injection of L-CYSme in particular elicited a substantial decrease in Te that remained lower than values in vehicle-treated rats for about 25 min. As shown in Panel C, the Ti/Te ratio fell markedly initially after injection of morphine, but rose substantially and remained elevated above baseline in both groups of rats. The injections of vehicle produced negligible responses, whereas the second but not first injection of L-CYSme caused a noticeable and sustained decrease in Ti/Te values in comparison to those in the vehicle-treated rats. As can be seen in Figure 3 (Panel A), the injection of morphine elicited a sustained increase in EIP in both groups of rats and that the injections of vehicle elicited negligible responses. However, the first injection of L-CYSme elicited substantial decreases in EIP that had returned to the sustained increase in EIP seen 15 min post morphine injection by the time the second injection was given. The second injection of L-CYSme produced a profound and long-lasting decrease in EIP. As can seen in Panel B, morphine elicited a pronounced transient increase in EEP that was followed by a sustained decrease in EEP. The two injections of L-CYSme produced trivial changes in EEP and did not alter the sustained decrease in EEP. Figure 4 (Panel A) demonstrates that morphine elicited a transient increase followed by a pronounced and sustained decrease in PIF, and that the injections of vehicle elicited minimal responses. In contrast, the injections of L-CYSme elicited pronounced and sustained increases in PIF with the first injection reversing the effects of morphine back to baseline values, and the second injection elevating PIF to values considerably above baseline values. As seen in Panel B, morphine elicited a transient reduction in PEF that had resolved by the time the injections of vehicle or L-CYSme were administered. The two injections of vehicle produced trivial changes in EEP, whereas the two injections of L-CYSme elicited pronounced and sustained increases in PEF to levels well above baseline values. Taken together, these changes in PIF and PEF resulted in morphine eliciting sustained decreases in PIF/PEF with the second injection of L-CYSme causing an increase towards baseline values, however this increase was not sustained over the 60 min recovery period. As seen in Figure 5 (Panel A), the injection of morphine elicited a transient increase that EF_50_ that was followed by a minor, but sustained, elevation in EF_50_ in the rats that received the two injections of vehicle. The two injections of L-CYSme elicited pronounced and sustained increases in EF_50_. As seen in Panel B, morphine elicited a transient decrease in RT that was followed by a sustained decrease in rats that received the two injections of vehicle. The second injection of L-CYSme elicited a pronounced further decrease in RT that had resolved by 60 min post-injection to values near the vehicle-treated rats. As summarized in Panel C, morphine elicited a transient but pronounced increase in apneic pause and the injections of both vehicle and L-CYSme elicited trivial responses. As shown in Figure 6, the injection of morphine elicited a prompt and sustained decrease in inspiratory drive (Panel A) and a pronounced, but relatively transient reduction in expiratory drive (Panel B). The injections of vehicle elicited trivial responses whereas the injections of L-CYSme produced pronounced and sustained increases in inspiratory and expiratory drives. As summarized in Figure 7, the injection of morphine elicited a transient increase in NEBI (Panel A) and NEBI/Freq (Panel B) that was followed by a sustained decrease in both parameters. Injections of L-CYSme elicited trivial responses and did not alter the time-course of morphine-induced changes in NEBI or NEBI/Freq observed in vehicle-injected rats. Figure 8 summarizes the total (cumulative) changes in ventilatory parameters recorded over the 15 min period after the first injection of vehicle or L-CYSme (expressed as %change from Pre values). The first injection of L-CYSme caused a sustained reversal of the deleterious effects of morphine on TV, MV, Ti, EIP and inspiratory drive (InspD). The first injection of L-CYSme also resulted in a minor increase in Freq and substantial increases in PEF and expiratory drive (ExpD), but did not alter the morphine-induced decreases in Te, EEP, PIF/PEF, NEBI or NEBI/Freq or the morphine-induced increases in Ti/Te. Figure 9 shows the total (cumulative) changes in ventilatory parameters recorded over the 60 min period after the second injection of vehicle or L-CYSme (%change from Pre values). The second injection of L-CYSme overcame the morphine-induced decreases in TV, MV, PIF and inspiratory drive (InspD) and slightly increased the morphine-induced decease in NEBI/Freq but not NEBI. The second injection of L-CYSme augmented the morphine-induced increase in EF_50_ and caused a substantial increase in Freq, PEF and expiratory drive (ExpD). The second injection of L-CYSme did not alter the morphine-induced decreases on Te, EEP, PIF/PEF or the lack of effects of morphine on apneic pause (AP).

**FIGURE 10:**
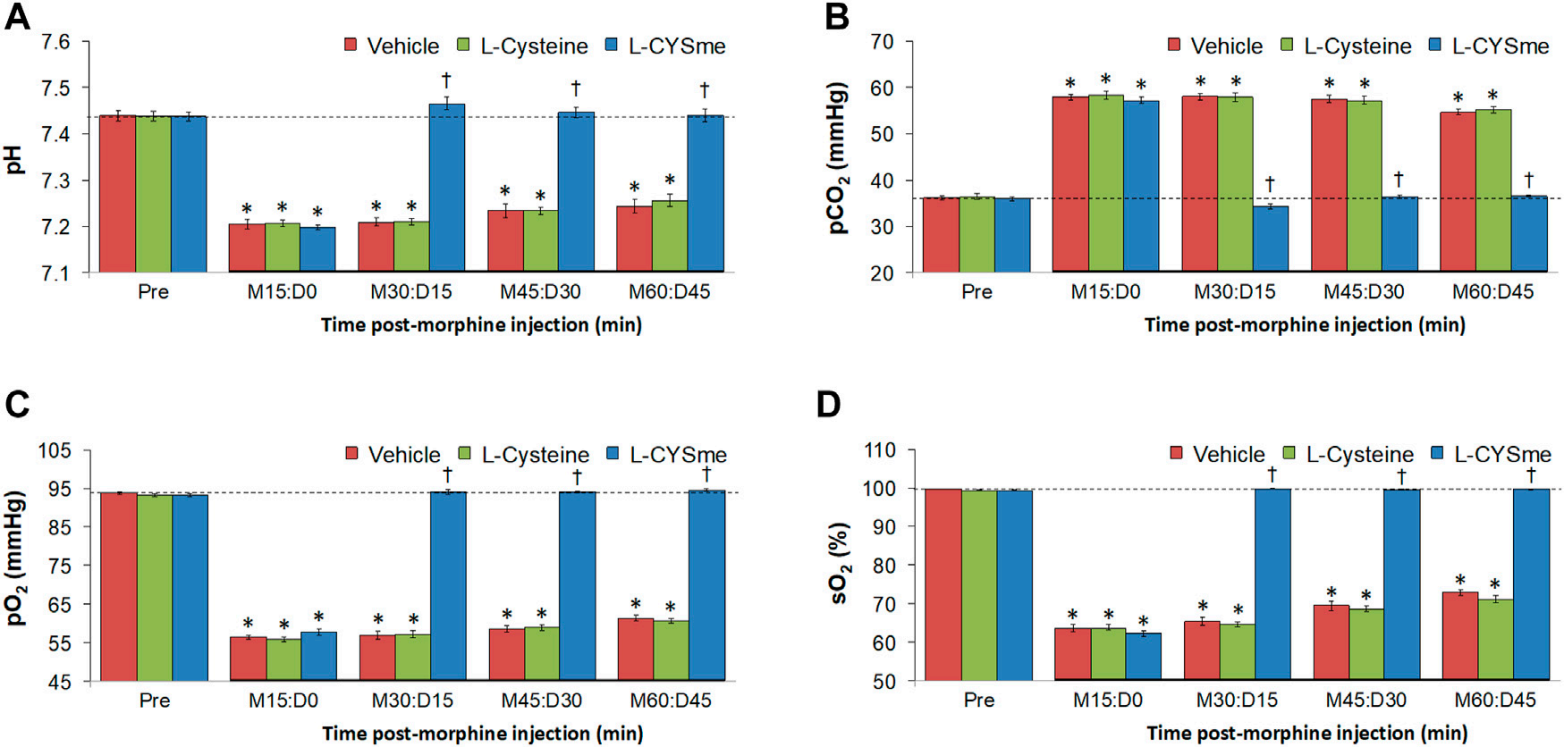
Values of pH Panel **(A)**, pCO_2_ Panel **(B)**, pO_2_ Panel **(C)** and sO_2_ Panel **(D)** before (Pre) and after injection of morphine (10 mg/kg, IV) in three separate groups of freely-moving rats followed by injection of vehicle (VEH, saline), L-cysteine (500 μmol/kg, IV) or L-cysteine methyl ester (L-CYSme, 500 μmol/kg, IV). The terms M15, M30, M30, M45 and M60 denote 15, 30, 45 and 60 min after injection of morphine. The terms D0, D15, D30 and D45 denote 0, 15, 30 and 45 min after injection of drug (vehicle, L-Cysteine or L-CYSme). The data are prsented as mean ± SEM. There were 9 rats in each group. *p < 0.05, significant change from Pre values. ^†^ *p* < 0.05, significant change between L-CYSme *versus* vehicle.

**FIGURE 11:**
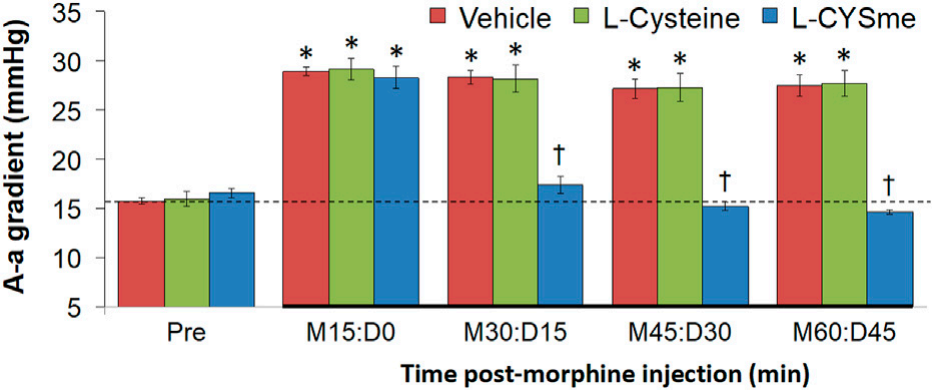
Alveolar-arterial (A-a) gradient values before (Pre) and after injection of morphine (10 mg/kg, IV) in three groups of freely-moving rats followed by injection of vehicle (VEH, saline), L-Cysteine (500 μmol/kg, IV) or L-cysteine methyl ester (L-CYSme, 500 μmol/kg, IV). The terms M15, M30, M45 and M60 denote 15, 30, 45 and 60 min after injection of morphine. The terms D0, D15, D30 and D45 denote 0, 15, 30 and 45 min after injection of drug (vehicle, L-Cysteine or L-CYSme). The data are mean ± SEM. There were 9 rats in each group. *p < 0.05, significant change from Pre values. ^†^ *p* < 0.05, significant change between L-CYSme *versus* vehicle.

The effects of L-cysteine or L-CYSme on morphine changes in ABG chemistry and A-a gradient are shown in Figure 10 summarizes the recorded ABG values (pH, pCO_2_, pO_2_ and sO_2_) before and after injection of morphine (10 mg/kg, IV) and subsequent injection of vehicle, L-cysteine (500 μmol/kg, IV) or L-CYSme (500 μmol/kg, IV) in three separate groups of unanesthetized rats. The values denoted M15 to M60 are the times post-morphine injection and the values denoted D0 to D45 reflect the times following injection of vehicle, L-cysteine or L-CYSme. Panel A shows that the injection of morphine elicited substantial and equivalent decreases in arterial blood pH in the three groups of rats and that the injection of L-CYSme, but not L-cysteine, elicited a prompt and sustained reversal of the acidosis. Panel B shows that injection of morphine produced a substantial and equivalent increase in pCO_2_ in the three treatment groups and that L-CYSme, but not L-cysteine, caused an immediate and sustained reversal of the hypercapnia. Panels C and D demonstrate that morphine produced sustained decreases in pO_2_ and sO_2_, respectively and that L-CYSme, but not L-cysteine, produced a prompt and sustained reversal of the hypoxemic status of the arterial blood. As summarized in Figure 11, the injection of morphine (10 mg/kg, IV) produced substantial and equivalent increases in A-a gradient values (index of diminished alveolar gas-exchange in the lungs) in the three groups of rats. As can be seen, the injection of L-CYSme (500 μmol/kg, IV), but not the injection of L-cysteine (500 μmol/kg, IV), produced an immediate and sustained reversal of this deleterious effect of morphine. The arithmetic changes in ventilatory parameters presented in Supplementary Table S6 reinforce all of the above conclusions.

### Effects of L-cysteine and L-CYSme on the antinociceptive actions of morphine

**FIGURE 12:**
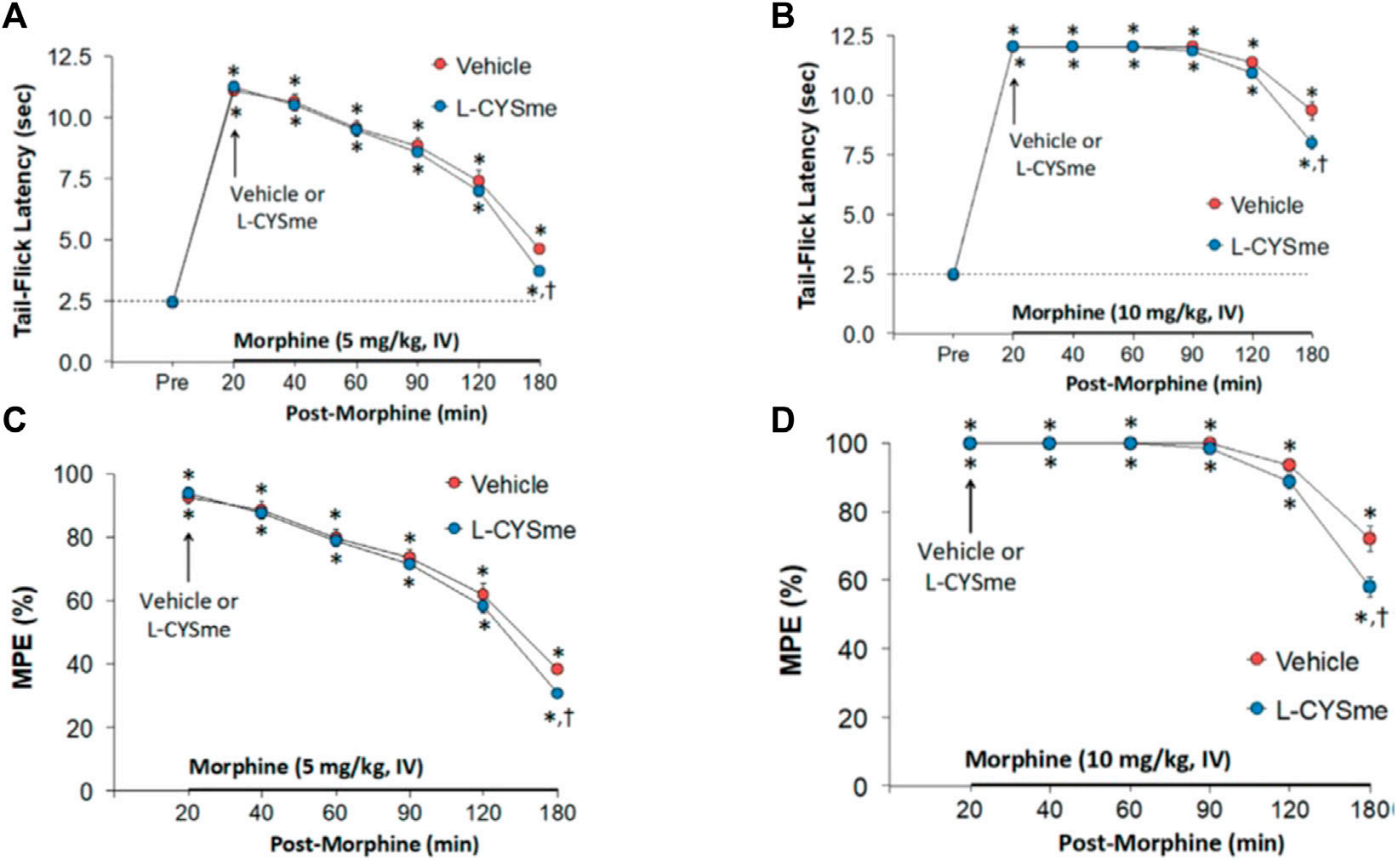
Changes in tail-flick latency values elicited by an intravenous injection of vehicle (VEH, saline) or L-cysteine methyl ester (L-CYSme, 500 μmol/kg, IV) given 20 min after the injection of morphine at 5 mg/kg Panel **(A)** or 10 mg/kg Panel **(B)** in freely-moving rats. Panels **(C,D)** display the data as maximum possible effect (%MPE). The data are shown as mean ± SEM. There were 6 rats in each group. *p < 0.05, significant change from Pre. ^†^ *p* < 0.05, significant change between L-CYSme *versus* vehicle.

As seen in Figure 12, the duration of the antinociceptive effects of morphine (TFL studies) were minimally reduced by the injection of L-CYSme (500 μmol/kg, IV) with differences only occurring at the 180 min time point. As seen in Supplementary Table S7, the injection of L-cysteine (500 μmol/kg, IV) elicited a transient decrease in TFL, whereas injections of L-CYSme (500 μmol/kg, IV) elicited a transient increase in TFL and HPL. All responses were evident 10 min after administration of L-cysteine or L-CYSme but not after 20 min, the time point when morphine (10 mg/kg, IV) was injected. Supplementary Figure S2 summarizes the magnitudes and durations of the antinociceptive effects of 5 mg/kg (Panels A and C) or 10 mg/kg (Panels B and D) doses of morphine as assessed by TFL assay in rats pre-treated with vehicle or L-CYSme (500 μmol/kg, IV). The duration of antinociception elicited by 10 mg/kg of morphine was greater than that elicited by the 5 mg/kg dose in the vehicle-treated rats. The duration, but not the magnitude, of the antinociceptive actions of morphine were diminished in L-CYSme-treated rats. The loss of antinociception began 120 min after injection of 5 mg/kg of morphine and 210 min after administration of the 10 mg/kg dose of the opioid. As summarized in Supplementary Figure S3, pre-treatment with L-CYSme (500 μmol/kg, IV) also reduced the duration, but not magnitude, of the antinociceptive actions of morphine (10 mg/kg, IV) as assessed by HPL assay. Again, the loss of antinociception began 120 min after the injection of 5 mg/kg dose of morphine and 210 min after injection of 10 mg/kg of the opioid. In contrast, neither the magnitude nor duration of antinociceptive actions of the 5 and 10 mg/kg doses of morphine were affected by pre-treatment with L-cysteine (Supplementary Figure S4).

### Effects of L-cysteine and L-CYSme and on morphine-induced sedation

The observed behaviors in each group of rats that received injections of morphine plus injections of vehicle, L-cysteine or L-CYSme were not obviously different to one another. More specifically, the injection of morphine elicited a relatively rapid (within 2–3 min) sedative effect in all of the rats that consisted as an almost complete loss of mobility and unusual body postures (see descriptions in Material and Methods). The full return of the modified righting-reflex in vehicle-treated rats (74.3 ± 11.5 min, n = 9), L-cysteine (500 μmol/kg, IV)-treated rats (69.8 ± 8.5 min, n = 9) and L-CYSme (500 μmol/kg, IV)-treated rats (91.0 ± 11.2 min, n = 9) were similar to one another (*p* > 0.05, for all between-group comparisons).

## Discussion

### Administration of L-CYSme overcame morphine-induced respiratory depression

The changes in ventilatory parameters elicited by a 10 mg/kg intravenous dose of morphine were consistent with those of our previous reports (May et al., 2013a; May et al., 2013b; Young et al., 2013; Baby et al., 2018; Baby S. M. et al., 2021; Gaston et al., 2021; Getsy et al., 2022d). Morphine elicited a transient reduction in Freq that was not truly reflective of its effects on respiratory timing because it elicited a sustained increase in Ti (i.e., a longer inspiratory duration), but a pronounced decrease in Te (i.e., a shorter expiratory duration). Moreover, morphine caused a long-lasting increase in EIP, whereas it elicited a transient increase followed by a sustained decrease in EEP. The ability of morphine to lengthen Ti while marginally affecting Te (Fone and Wilson, 1986; Kasaba et al., 1997; Chevillard et al., 2009) or the shortening of Te (May et al., 2013a; May et al., 2013b; Young et al., 2013; Baby et al., 2018; Baby S. M. et al., 2021; Gaston et al., 2021) is well-known, as is the propensity of opioids to differentially affect EIP and EEP (May et al., 2013a; May et al., 2013b; Henderson et al., 2013; Young et al., 2013; Henderson et al., 2014; Baby et al., 2018; Baby S. M. et al., 2021; Gaston et al., 2021). Mechanisms of action and brain structures, including nucleus tractus solitarius (Hassen et al., 1982; Li et al., 1996; Iniushkin, 1997; Zhuang et al., 2012), parabrachial nucleus/Kölliker-Fuse nucleus (Eguchi et al., 1987; Lalley et al., 2014; Bachmutsky et al., 2020; Varga et al., 2020), and pre-Bötzinger complex (Bachmutsky et al., 2020; Varga et al., 2020), by which morphine and fentanyl affect inspiratory and expiratory timing have been investigated. It is evident that the qualitative and quantitative responses elicited by opioids, such as morphine, fentanyl and remifentanil, on Ti and Te are very much dose-dependent (Li et al., 1996; Henderson et al., 2013; Henderson et al., 2014; Palkovic et al., 2020; Palkovic et al., 2021; Palkovic et al., 2022). However, the sites and mechanisms by which opioids exert their differential effects on EIP and EEP have received only limited attention (Henderson et al., 2014; Bachmutsky et al., 2020). Studies in anesthetized rats and *in vitro* preparations found that depression of carotid body chemoreceptor afferent reflexes participates in morphine-induced reduction in Freq, whereas morphine (10 mg/kg)-induced reduction in Freq is enhanced in *unanesthetized* Sprague Dawley rats with bilateral carotid sinus nerve transection (Baby et al., 2018), suggesting that morphine augments chemoreflex activity in unanesthetized rats. Morphine elicited long-lasting decreases in TV, MV and PIF and RT, a long-lasting increase in EF_50_, a transient decrease in PEF, a substantial, but transient, increase in apneic pause, and an initial increase and then a sustained decrease in NEBI (May et al., 2013a; May et al., 2013b; Young et al., 2013; Baby et al., 2018; Baby S. M. et al., 2021; Gaston et al., 2021; Getsy et al., 2022d). This multi-directional pattern of ventilatory responses points to the disparate role of OR signaling mechanisms in the control of breathing. How quickly effective opioid concentrations reach the multiple target sites will obviously play a major role in the temporal and dose-dependent effects of opioids ventilation. expression. This rate will depend on the (1) plasma concentration, (2) lipophilicity/ability to cross the blood brain barrier (fentanyl crosses the blood-brain barrier faster than morphine), (3) diffusion time to neurons of interest, and (4) synaptic concentrations required to affect different neuron subtypes (Haji et al., 2003). Moreover, Haji et al. (2003) provided compelling evidence that morphine depresses respiratory neuronal activity through two different intracellular mechanisms, both of which are elicited by μ-ORs.

L-CYSme (500 μmol/kg) elicited a rapid reversal of the adverse effects of morphine (10 mg/kg) on ventilation in unanesthetized rats. L-CYSme overcame the effects of morphine on TV, MV, Ti, EIP, inspiratory and expiratory drives, elicited pronounced increases in Freq (at a time when the initial depressant effects of, morphine on Freq had mostly subsided), augmented morphine-induced decreases in Te, and augmented morphine-induced increases in EF_50_, but did not affect morphine-induced decreases in EEP, RT, NEBI or NEBI/Freq. The first injection of L-CYSme overcame most of the deleterious effects of morphine, whereas the second injection often produced dramatic changes in ventilation. These findings add to knowledge of L-CYSme, including the oxidation of L-CYSme to L-cystine dimethylester (Krämer and Schmidt, 1984), the formation of mixed disulfides with lipoic acid (Ishii et al., 2010), alterations in mechanical properties of dipalmitoyl-phosphatidyl-choline in plasma membranes (Arias et al., 2019), hydrogen bonding to chloride ions (Mosier-Boss and Lieberman, 2005), interactions with myoglobin (Shimizu et al., 1976), redox reduction of cytochrome C (Engman et al., 1994), altered stress-induced changes in cardiac intracellular Ca^2+^ (Fukui et al., 1994), formation of or one-electron reduction of S-nitrosothiols (Liu et al., 1994; Manoj et al., 2006; Hu and Ho, 2011), regulation of aminotransferases (Pagani et al., 1994), non-enzymatic isomerization of 9-cis-retinoic acid (Shih et al., 1997), disassembly/reassembly of [2Fe-2S] clusters in redox-regulated transcription factor SoxR (Ding and Demple, 1998), the ability to act as substrates for or be oxidized by peroxidases (Svensson, 1988a; Svensson, 1988b; Svensson et al., 1993; Burner et al., 1999), potentiation of glucose-induced insulin release (Ammon et al., 1986), the ability to reduce mucus viscosity (Yanaura et al., 1982), the ability to enter into lungs (Hobbs et al., 1993; Hobbs et al., 1998; Lalley, 2004), and the ability to reduce pulmonary edema (Lailey et al., 1991). We have not determined mechanisms by which L-CYSme exerts its effects against morphine although the lack of immediate effects of L-CYSme on the analgesic/sedative effect of morphine suggest that unlike OR antagonists (van Dorp et al., 2007; Dahan et al., 2010; Boom et al., 2012; Dahan et al., 2018; Algera et al., 2019), the L-thiolester does not block ORs by competitive or allosteric binding. Moreover, establishing that L-cysteine (500 μmol/kg, IV) did not modify any of the actions of morphine suggests that intracellular entry is key to the activity of L-CYSme and that upon entry it exerts its effects as the thiolester itself, or de-esterification to L-cysteine and formation of bioactive L-cysteine derivatives, such as hydrogen sulfide (Wu et al., 2009; Yin et al., 2016; Paul et al., 2018), cysteine-sulfenic, -sulfinic and -sulphonic acids (Yamaguchi and Hosokawa, 1987; Joseph and Maroney, 2007; Stipanuk et al., 2009; Stipanuk et al., 2011) and the S-nitrosothiol, S-nitroso-L-cysteine (Myers et al., 1990; Bates et al., 1991; Foster et al., 2009; Seth and Stamler, 2011; Gaston et al., 2020; Seckler et al., 2020). The lack of effect of L-cysteine may also be due to the ability of morphine to inhibit L-cysteine uptake into neurons via inhibition of excitatory amino acid transporter type 3 (Trivedi et al., 2014; Trivedi and Deth, 2015) although it is unknown whether morphine affects other L-cysteine up-take systems (Tunnicliff, 1994; Shanker and Aschner, 2001; Aoyama et al., 2008; Albrecht and Zielińsk, 2019), including excitatory amino acid transporters 1 and 2 (Zerangue and Kavanaugh, 1996; Chen and Swanson, 2003; Himi et al., 2003; Trivedi et al., 2014; Trivedi and Deth, 2015; Albrecht and Zielińsk, 2019), large neutral amino acid transporters (Simmons-Willis et al., 2002; Nemoto et al., 2003; Li and Whorton, 2007; Granillo et al., 2008), band three protein-anion transport system (Young et al., 1981; Tunnicliff, 1994) and high affinity Na^+^-dependent glutamate transporters (Hayes et al., 2005).

With respect to where L-CYSme may act to overcome the deleterious effects of morphine on ventilatory parameters, intravenous injection (10 mg/kg, 100 μCi/kg) of ^35^S-L-CYSme (Wepierre et al., 1964) or ^35^S-L-CYSee (Servin et al., 1988) resulted in rapid appearance in all tissues, organs and blood. At 5 min, ^35^S-L-CYSme and ^35^S-L-CYSee were highest in lungs, kidneys, chest-wall muscle, intestines and brain (only ^35^S-L-CYSee studied in the brain). Low levels of ^35^S-L-CYSme and ^35^S-L-CYSee were observed in blood, likely reflecting rapid dispersal of L-thiolesters into tissues. After 60 min, ^35^S-L-CYSme and ^35^S-L-CYSee were heavily seen in lungs, chest-wall, liver, kidneys and intestines, but diminished in brain with a few regions showing strong labeling. In contrast, intravenous injection (10 mg/kg, 100 μCi/kg) of ^35^S-L-cysteine resulted in intense labeling in kidneys and liver, but not lungs, chest-wall muscle or brain (Servin et al., 1988). As such, L-CYSme may enter central and peripheral structures that mediate the effects of morphine on breathing (Pattinson, 2008; Boom et al., 2012; Henderson et al., 2013; Henderson et al., 2014; Palkovic et al., 2020) thereby overcoming signaling events eliciting OIRD. A second injection of L-CYSme (500 μmol/kg) elicited pronounced/sustained effects on breathing including rises in Freq, TV and MV. These effects contrast those seen with D-CYSee since injection of the D-thiolester (500 μmol/kg) caused an immediate and sustained reversal of the effects of morphine (10 mg/kg) on ventilation, but a second injection elicited minor additional responses (Getsy et al., 2022d). It would appear that higher levels of L-CYSme activate functional proteins and/or enter into metabolic pathways not available to D-CYSee. The findings with L-CYSme add to evidence regarding the efficacy of reduced (monosulfide) thiolesters, such as L-GSHee (Jenkins et al., 2021) and D-CYSee (Getsy et al., 2022c; Getsy et al., 2022d), and oxidized (disulfide) thiolesters, such as D-CYSdiee and D-CYSdime (Gaston et al., 2021) in preventing/overcoming the deleterious actions of morphine or fentanyl on ventilation. The possibility that the mechanisms of action of L-CYSme involves formation of S-nitrosyl forms of L-CYSme and/or L-cysteine is supported by our findings that the ability of morphine or fentanyl to adversely affect ventilation, ABG chemistry and A-a gradient are markedly reduced in rats receiving continuous intravenous infusions of SNO-L-cysteine (Getsy et al., 2022a; Getsy et al., 2022b), whereas the adverse effects of fentanyl (morphine not studied to date) are augmented after inhibition of nitric oxide synthase (Seckler et al., 2022). It is well-studied that SNO-L-cysteine regulates many intracellular signaling cascades (Lipton et al., 1993; Foster et al., 2009; Seth and Stamler, 2011; Stomberski et al., 2019; Gaston et al., 2020), including cardiorespiratory control systems (Davisson et al., 1996; Davisson et al., 1997; Ohta et al., 1997; Lipton et al., 2001; Lewis et al., 2006; Gaston et al., 2020) and those involved in attenuation of OIRD (Getsy et al., 2022a; Getsy et al., 2022b).

### Administration of L-CYSme overcame morphine-induced changes in ABG chemistry and A-a gradient

Consistent with previous findings (Baby et al., 2018; Baby S. M. et al., 2021; Gaston et al., 2021; Getsy et al., 2022a; Getsy et al., 2022c; Getsy et al., 2022d), morphine (10 mg/kg, IV) elicited pronounced and long-lasting adverse changes in ABG chemistry in unanesthetized rats that consisted of a decrease in arterial blood pH, an increase in pCO_2_, and decreases in pO_2_ and sO_2_, responses consistent with morphine hypoventilation. These changes in ABG chemistry were associated with sustained increases in A-a gradient, suggesting that morphine adversely affected alveolar gas-exchange (Gaston et al., 2021; Getsy et al., 2022a; Getsy et al., 2022c; Getsy et al., 2022d). The elevation in A-a gradient could signify atelectasis (i.e., alveolar collapse) due to hypoventilation, but may also involve more complicated effects on surfactant status/alveolar fluid clearance, which if adversely affected would impair alveolar gas-exchange, despite the morphine-induced decrease in TV. The principal end-result of any therapeutic being developed to overcome OIRD must be restoration of normal ABG chemistry, and so the ability of the therapeutic to drive breathing should not be compromised by untoward effects on other functions driving breathing or gas-exchange. The second important set of findings was that a single injection of L-CYSme (500 μmol/kg, IV), but not L-cysteine (500 μmol/kg, IV), elicited an immediate and sustained reversal of the adverse effects of a 10 mg/kg dose of morphine on ABG chemistry and A-a gradient. As such, the ability of L-CYSme to overcome the effects of morphine on ventilation are not compromised by untoward effects on the upper airway and/or gas exchange as produced by L-CYSee in isoflurane-anesthetized rats (Mendoza et al., 2013), remembering that the co-administration of L-CYSee and morphine does not adversely affect the upper airway in rats anesthetized with sevoflurane (Bates et al., 1991 unpublished findings). Although the abilities of L-CYSme, D-CYSee (Getsy et al., 2022c; Getsy et al., 2022d) and L-CYSee (Mendoza et al., 2013) to overcome the effects of morphine on ventilation and alveolar gas-exchange may involve supply of intracellular reducing equivalents, we found that the powerful reducing/cell-permeable L-thiolester, N-acetyl-L-cysteine methylester (L-NACme) (Tsikas et al., 2018), had minor effects on the ability of morphine to depress ventilation (Gaston et al., 2021). The rapid conversion of L-NACme to L-cysteine in cells (Lailey and Upshall, 1994) argues that the beneficial actions of L-CYSme on morphine OIRD are not due to provision of reducing equivalents in cells or entry of L-cysteine into metabolic pathways. Rather, these findings suggest that the efficacy of L-CYSme, L-GSHee (Jenkins et al., 2021), D-CYSee (Getsy et al., 2022c; Getsy et al., 2022d) and D-cystine di(m)ethylester (Gaston et al., 2021) may involve the intracellular actions of the thiolester moieties themselves. Our findings that intravenous Tempol, a scavenger of free-radicals/superoxide anion, attenuates OIRD elicited by morphine or fentanyl (Baby S. et al., 2021; Baby S. M. et al., 2021) points to the importance of redox mechanisms in opioid actions and possibly the ability of thiolesters to overcome OIRD. The effectiveness of L-CYSme to overcome the adverse effects of morphine in unanesthetized rats and in sevoflurane-anesthetized rats, the most clinically-relevant gaseous anesthetic (Aoki et al., 2021; Apai et al., 2021; Liang et al., 2021), supports the necessity of further studies with L-CYSme in pre-clinical models of OIRD, such as dogs (Walker et al., 1967; Lemmens et al., 2008) or goats (Hubbard et al., 1992; Heard et al., 1996) that bridge potential clinical trials studies in humans.

### L-CYSme-induced changes in morphine-induced antinociception

Intravenous doses of 5 or 10 mg/kg of morphine elicited robust antinociception for over 180 and 240 min in TFL and HPL assays. The antinociception actions of morphine were reduced 160 min post-L-CYSme (180 min post-morphine) as compared to vehicle controls. The 500 μmol/kg dose of L-CYSme in naïve rats increased TFL and HPL at 10 min but not at 20 min. Because no behaviors occurred upon injection, L-CYSme may have elicited short-lived antinociception. The antinociceptive effects of morphine given after L-CYSme were also diminished in duration (evident 2.5 h after 5 mg/kg of morphine and 3.5 h after 10 mg/kg morphine). In contrast, 500 μmol/kg of L-cysteine did not affect TFL, HPL or the actions of morphine. L-cysteine enters cells by several uptake systems (Young et al., 1981; Tunnicliff, 1994; Zerangue and Kavanaugh, 1996; Shanker and Aschner, 2001; Simmons-Willis et al., 2002; Chen and Swanson, 2003; Himi et al., 2003; Nemoto et al., 2003; Hayes et al., 2005; Li and Whorton, 2007; Aoyama et al., 2008; Granillo et al., 2008; Trivedi et al., 2014; Trivedi and Deth, 2015; Albrecht and Zielińsk, 2019). As such, any effects of L-cysteine may have occurred before the 10 min testing time or not enough L-cysteine entered into cells affecting antinociceptive processing. The effects of microinjections of L-cysteine, D-cysteine and L- and D-cystine into the hindpaw (Todorovic et al., 2001; Todorovic et al., 2004; Nelson et al., 2005; Pathirathna et al., 2006; Nelson et al., 2010; Todorovic and Jevtovic-Todorovic, 2014), and sensory cell bodies in dorsal root ganglia (Jagodic et al., 2007) and thalamus (Joksovic et al., 2006) on antinociception in rats have been studied. L-cysteine was pronociceptive, whereas L-cystine was antinociceptive (Todorovic et al., 2001; Todorovic et al., 2004; Nelson et al., 2005; Joksovic et al., 2006; Pathirathna et al., 2006; Jagodic et al., 2007; Nelson et al., 2010; Todorovic and Jevtovic-Todorovic, 2014). Direct intra-dermal microinjections of L-cysteine into the peripheral receptive field (ventral side) of the right hind paw of rats elicited a dose/time-dependent hyperalgesia that was prevented by a neuroactive steroid, 3-βOH, which blocks voltage-dependent T-type Ca^2+^ channels (Pathirathna et al., 2006). Moreover, L-cysteine caused hyperalgesia upon microinjection into the thalamus via activation of CaV_3.2_ (Joksovic et al., 2006). The lack of effects of L-cysteine in the present study may be due to lack of rapid/sufficient entry into cells, whereas L-CYSme antinociception may involve extracellular/intracellular actions of the L-thiolester moiety. The initial antinociception responses elicited by 5 and 10 mg/kg morphine were not reduced by L-CYSme suggesting that the L-thiolester did not block ORs in central and/or peripheral pathways that elicit opioid antinociception (Connor and Christie, 1999; Williams et al., 2013; Henderson et al., 2014). However, the decrease in the duration of the antinociceptive actions of morphine by L-CYSme suggests that this L-thiolester does enter these particular pathways to gradually establish intracellular processes that countermand morphine antinociception. L-CYSme may exert antinociceptive actions as the L-thiolester whose effects are counterbalanced by de-esterification to L-cysteine, which as its levels rise, gradually overcomes morphine antinociception by activating voltage-dependent T-type Ca^2+^ channels (Joksovic et al., 2006; Pathirathna et al., 2006). S-nitroso-L-cysteine *diminishes* T-type Ca^2+^ channel activity upon microinjection into the thalamus of rats (Joksovic et al., 2007; Lee et al., 2013). Therefore, L-CYSme may be S-nitrosylated upon entry into nociceptive-antinociceptive pathways to promote morphine antinociception. As the levels of S-nitroso-L-CYSme decrease over time and those of L-cysteine increase, the nociceptive effects of L-cysteine would dominate those of SNO-L-CYSme thereby reducing morphine antinociception.

### Study limitations

A limitation of the present study is that we have not tested the efficacy of lower doses of L-CYSme on morphine-induced OIRD. Lower doses may prove effective against OIRD and have less effect on the duration of morphine antinociception. Our evidence that 500 μmol/kg of L-CYSme overcomes the adverse effects of morphine on breathing, ABG chemistry and gas-exchange in alveoli, should be extended to include whether 100 or 250 μmol/kg doses overcome morphine OIRD. Synthetic opioids are playing a major role in the current opioid crisis (Arendt, 2021; Deo et al., 2021) and further investigation needs to include whether L-CYSme can overcome the adverse actions of high potency opioids, such as fentanyl. Another limitation of this study is the lack of data about the efficacy of L- or D-thiolesters in preventing/reversing OIRD in female rats, especially since opioids exert qualitatively and quantitatively different ventilatory responses in females compared to males (Dahan et al., 1998; Hosseini et al., 2011). Yet another limitation is that full dose-response studies with L-CYSme or other L-,D-thiolesters have not been performed that would allow determination of a maximally effective dose, maximal duration of action, and also potentially limiting side-effects in unanesthetized rats. At present, it is unknown whether the different magnitudes of effects of L-CYSme compared to the thiolester, D-CYSee, for example, depend on drug kinetics rather than on efficacy.

Additionally, the lack of understanding about the molecular mechanisms by which L-CYSme affects morphine- and/or fentanyl-induced OIRD also presents a limitation to this study. Aside from possible direct interactions with yet to be determined functional proteins, potential mechanisms of action of L-CYSme may involve (1) direct binding to putative L-, D-cysteine binding protein, a myristoylated alanine-rich C-kinase substrate (Semenza et al., 2021), (2) interruption of OR-β-arrestin-coupled cell signaling processes to spare the antinociceptive G-protein-dependent actions of morphine (Schmid et al., 2017; Grim et al., 2020), and/or (3) potential conversion of L-CYSme to S-nitroso-L-CYSme or S-nitroso-L-cysteine, by S-nitrosylation of the sulfur atom in L-thiolesters via processes requiring nitric oxide synthase (Perissinotti et al., 2005; Hess and Stamler, 2012; Stomberski et al., 2019; Seckler et al., 2022), which may act in a similar way to the intracellular penetrating L-thiolester, S-nitroso-L-cysteine ethyl ester (Clancy et al., 2001). To test the possibility that L-CYSme elicits the production of S-nitrosylated versions of the L-thiolester, we are determining whether intravenous injections of L-CYSme increase production of S-nitrosylated species in blood, peripheral tissues, and brain via the use of an ultra-sensitive capacitive sensor (Seckler et al., 2017) and whether such injections of L-CYSme increase expression of NADPH diaphorase in the brain and peripheral structures, on the basis that NADPH diaphorase visualizes free S-nitrosothiols and S-nitrosylated proteins in aldehyde-treated tissue (Seckler et al., 2020). It is well-known that S-nitrosothiols, such as S-nitroso-L-cysteine and S-nitroso-L-glutathione, play important roles in ventilatory control processes within the brainstem, circulating red blood cells, and peripheral structures, such as the carotid bodies (Gaston et al., 2001; Lipton et al., 2001; Palmer et al., 2013; Gaston et al., 2014; Palmer et al., 2015; Gaston et al., 2020). The ability of morphine or fentanyl to depress breathing and adversely affect ABG chemistry is substantially reduced in rats receiving intravenous infusion of S-nitroso-L-cysteine (Getsy et al., 2022a; Getsy et al., 2022b). The efficacy of S-nitroso-L-cysteine adds to our knowledge regarding the ability of L-S-nitrosothiols to profoundly affect cardiorespiratory control systems (Davisson et al., 1996; Lewis et al., 1996; Travis et al., 1996; Davisson et al., 1997; Ohta et al., 1997; Travis et al., 1997; Gaston et al., 2001; Lewis et al., 2005; Lewis et al., 2006; Gaston et al., 2020). We are currently determining the degree that systemic injections of L-CYSme generate S-nitrosothiols within key cardiorespiratory control brain structures, including the nucleus tractus solitarius, retrotrapezoid nucleus, Kölliker-Fuse nucleus, pre-Bötzinger complex, and key peripheral structures, such as the carotid bodies, diaphragm, chest-wall, and larynx, employing sensor technology (Seckler et al., 2017) and NADPH diaphorase histochemistry (Seckler et al., 2020). To address our lack of understanding of the temporal distribution of L-CYSme in blood and tissues, we are doing pharmacokinetics studies using liquid chromatography-mass spectrometry (Altawallbeh et al., 2019) to determine temporal distributions of L-CYSme in blood, peripheral structures, cerebrospinal fluid, and brain structures relevant to expression of OIRD, including the medulla oblongata, pons and medial prefrontal cortex. We are also testing whether L-CYSme ameliorates the latent adverse effects of morphine on ventilatory responses to hypoxic and hypoxic-hypercapnic (May et al., 2013a; May et al., 2013b) exposures. An important concern is whether the efficacies of OIRD reversal agents in rats will translate into therapies that overcome OIRD in humans. Many such OIRD reversal agents that have demonstrated efficacy in rats lacked such efficacy in human clinical trials (Dahan et al., 2018; Algera et al., 2019). A true limitation also pertains to our lack of understanding as to how L-CYSme reduces the duration of morphine antinociception. The potential mechanisms of action of L-CYSme described above provide us with starting points to explore the mechanisms by which these affects of L-CYSme are exerted.

## Conclusion

This study demonstrates that systemic injection of L-CYSme overcame the adverse effects of morphine on breathing, A-a gradient, and ABG chemistry in unanesthetized, unrestrained male Sprague Dawley rats. L-CYSme did not diminish the duration of morphine sedation, whereas it did decrease the duration of morphine antinociception. Since L-cysteine did not exert similar effects to those of L-CYSme, we propose that L-CYSme may overcome the deleterious effects of morphine on ventilation by mechanisms other than direct antagonism of ORs by processes including reversal of OR-initiated intracellular signaling processes and/or by increasing the activity of neurons that were not directly affected by opioids, but which contribute excitatory drive to the respiratory pattern generator. It is possible that administration of L-CYSme may result in a gradual appearance of compounds that overcome the effects of morphine or act as algesic substances. We have not investigated these possibilities, but know that thiols, such as mercaptoethanol, form conjugates with the minor morphine metabolite, morphinone (Yamano et al., 1985). Whether conjugate formation or potential ability of L-thiolesters to augment enzymatic conversion of morphine to major metabolites, such as morphine-3-glucuronide (Blomqvist et al., 2020), contribute to how L-CYSme overcomes the adverse effects of morphine is unknown. It is likely that the ability of L-CYSme to enter the brain and spinal cord likely overcomes the effect of morphine on breathing although peripheral mechanisms may also be involved since peripherally-restricted OR antagonists reduce OIRD (Lewanowitsch and Irvine, 2002; Lewanowitsch et al., 2006; Henderson et al., 2014). Our results with L-CYSme add to knowledge about the efficacy of L,D-thiolesters, such as L-GSHee (Jenkins et al., 2021), D-CYSee (Getsy et al., 2022c; Getsy et al., 2022d), D-cystine di(m)ethylester (Gaston et al., 2021) and the free radical-superoxide anion scavenger, Tempol, against OIRD (Baby S. et al., 2021; Baby S. M. et al., 2021).

## Data Availability

The raw data supporting the conclusions of this article will be made available by the authors, without undue reservation.

## Abbreviations:

ABG, arterial blood-gas chemistry; A-a gradient, Alveolar-arterial gradient; BK channel, large conductance Ca2+-activated K+ channel; D-CYSdiee, D-cystine diethylester; D-CYSdime, D-cystine dimethyl ester; EIP, end inspiratory pause; EEP, end expiratory pause; EF50, expiratory flow at 50% expired TV; D-CYSee, D-cysteine ethylester; EAA1-3, type 1–3 excitatory amino acid transporters; freq, frequency of breathing; L-CYSme, L-cysteine methylester; L-CYSee, L-cysteine ethylester; y-L-GCee, L-glutamyl cysteine ethylester; L-GSHee, L-glutathione ethylester; MV, minute ventilation; NEBI, non-eupneic breathing index; OR, opioid receptor; PIF, peak inspiratory flow; PEF, peak expiratory flow; RT, relaxation time; TV, tidal volume; Ti, inspiratory time; Te, expiratory time.
