# Supplementary material for: L-cysteine methyl ester overcomes the deleterious effects of morphine on ventilatory parameters and arterial blood-gas chemistry in unanesthetized rats
Source: Front Pharmacol. 2022 Sep 28;13:968378. doi: 10.3389/fphar.2022.968378 (PMC9554613; doi:10.3389/fphar.2022.968378)
Supplement: Supplementary file 1 [file DataSheet1.docx]

**Supplementary File**

**L-cysteine methyl ester overcomes the deleterious effects of morphine on ventilatory parameters and arterial blood-gas chemistry in unanesthetized rats**

Paulina M. Getsy,^1^* Santhosh M. Baby,^2,†^ Walter J. May,^3^ James N. Bates,^4^ Christopher R. Ellis,^5^ Michael G. Feasel,^5^ Christopher G. Wilson,^6^ Tristan H.J. Lewis,^1^ Benjamin Gaston,^7^ Yee-Hsee Hsieh,^8^

and Stephen J. Lewis^1,9^

*^1^Department of Pediatrics, Case Western Reserve University, Cleveland, OH 44106, USA*

*^2^Galleon Pharmaceuticals, Inc., 213 Witmer Road, Horsham, PA 19044, USA*

*^3^Pediatric Respiratory Medicine, University of Virginia School of Medicine,*

*Charlottesville, VA 22908, USA*

*^4^Department of Anesthesiology, University of Iowa Hospitals and Clinics, Iowa City, IA 52242, USA*

*^5^*United States Army CCDC Chemical Biological Center, Aberdeen Proving Ground, MD 21005, USA

*^6^Basic Sciences, Division of Physiology, School of Medicine, Loma Linda University,*

*Loma Linda, CA 92350, USA.*

*^7^Herman B Wells Center for Pediatric Research, Indiana University School of Medicine,*

*Indianapolis, IN 46202, USA*

*^8^Division of Pulmonary, Critical Care and Sleep Medicine, Case Western Reserve University,*

*Cleveland, OH 44106, USA*

*^9^Department of Pharmacology, Case Western Reserve University, Cleveland, OH 44106, USA*

**^†^Current address:** Santhosh M. Baby, Translational Sciences Treatment Discovery, Galvani Bioelectronics, Inc., 1250 S Collegeville Rd., Collegeville, Pennsylvania 19426. Email: santhosh.m.baby@galvani.bio

**Corresponding Author:** Paulina M. Getsy, PhD. Department of Pediatrics, Division of Pulmonology, Allergy and Immunology. School of Medicine. Case Western Reserve University, 10900 Euclid Avenue, Cleveland, OH 44106-4984. Phone: 216-368-5115. Email: pxg55@case.edu

**Supplementary Table S1.** Classes of drugs that may overcome opioid-induced respiratory depression

| **K^+^-channel blockers** |
| --- |
| Sia, R.L., and Zandstra, D.F. (1981). 4-Aminopyridine reversal of fentanyl-induced respiratory depression in normocapnic and hypercapnic patients. *Br. J. Anaesth*. 53, 373-379. doi: 10.1093/bja/53.4.373  Roozekrans, M., van der Schrier, R., Okkerse, P., Hay, J., McLeod, J.F., Dahan, A. (2014). Two studies on reversal of opioid-induced respiratory depression by BK-channel blocker GAL021 in human volunteers. *Anesthesiology* 121, 459-468. doi: 10.1097/ALN.0000000000000367  Golder, F.J., Dax, S., Baby, S.M., Gruber, R., Hoshi, T., Ideo, C., Kennedy, A., Peng, S., Puskovic, V., Ritchie, D., Woodward, R., Wardle, R.L., Van Scott, M.R., Mannion, J.C., and MacIntyre, D.E. (2015). Identification and Characterization of GAL-021 as a Novel Breathing Control Modulator. *Anesthesiology* 123, 1093-1104. doi: 10.1097/ALN.0000000000000844.  Roozekrans, M., Olofsen, E., van der Schrier, R., van Gerven, J., Peng, S., McLeod J., and Dahan, A. (2015). Reversal of opioid-induced respiratory depression by BK-channel blocker GAL021: A pharmacokinetic-pharmacodynamic modeling study in healthy volunteers. *Clin. Pharmacol. Ther*. 97, 641-649. doi: 10.1002/cpt.99  Wei, A.D., and Ramirez, J.M. (2019). Presynaptic Mechanisms and KCNQ Potassium Channels Modulate Opioid Depression of Respiratory Drive. *Front. Physiol*. 10, 1407. doi: 10.3389/fphys.2019.01407 |
| **Acetylcholinesterase enzyme inhibitors** |
| Elmalem, E., Chorev, M., and Weinstock, M. (1991). Antagonism of morphine-induced respiratory depression by novel anticholinesterase agents. *Neuropharmacology* 30, 1059-1064. doi: 10.1016/0028-3908(91)90134-w  Tsujita, M., Sakuraba, S., Kuribayashi, J., Hosokawa, Y., Hatori, E., Okada, Y., Kashiwagi, M., Takeda, J., and Kuwana, S. (2007). Antagonism of morphine-induced central respiratory depression by donepezil in the anesthetized rabbit. *Biol. Res*. 40, 339-346.  Sakuraba, S., Tsujita, M., Arisaka, H., Takeda, J., Yoshida, K., and Kuwana, S. (2009). Donepezil reverses buprenorphine-induced central respiratory depression in anesthetized rabbits. *Biol. Res*. 42, 469-475. |
| **α_2_-adrenoceptor antagonism** |
| Vonhof, S., and Sirén, A.L (1991). Reversal of μ-opioid-mediated respiratory depression by α2-adrenoceptor antagonism. *Life Sci*. 1991; 49, 111-119. doi: 10.1016/0024-3205(91)90024-6 |
| **Phosphodiesterase inhibitors** |
| Kasaba, T., Takeshita, M., and Takasaki, M. (1997). The effects of caffeine on the respiratory depression by morphine. *Masui* 46, 1570-1574.  Kimura, S., Ohi, Y., and Haji, A. (2015). Blockade of phosphodiesterase 4 reverses morphine-induced ventilatory disturbance without loss of analgesia. *Life Sci*. 127, 32-38. doi: 10.1016/j.lfs.2015.02.006 |
| **Adenylate cyclase (cAMP producing) activator forskolin** |
| Ballanyi, K., Lalley, P.M, Hoch, B., and Richter, D.W. (1997). cAMP-dependent reversal of opioid- and prostaglandin-mediated depression of the isolated respiratory network in newborn rats. *J. Physiol*. 504, 127-134. doi: 10.1111/j.1469-7793.1997.127bf.x |
| **Dopamine DA1 receptor agonists** |
| Ballanyi, K., Lalley, P.M, Hoch, B., and Richter, D.W. (1997). cAMP-dependent reversal of opioid- and prostaglandin-mediated depression of the isolated respiratory network in newborn rats. *J. Physiol*. 504, 127-134. doi: 10.1111/j.1469-7793.1997.127bf.x  Lalley, P.M. (2004). Dopamine1 receptor agonists reverse opioid respiratory network depression, increase CO_2_ reactivity. *Respir. Physiol. Neurobiol*. 139, 247-262. doi: 10.1016/j.resp.2003.10.007  Lalley, P.M. (2005). D1-dopamine receptor agonists prevent and reverse opiate depression of breathing but not antinociception in the cat. *Am. J. Physiol. Regul. Integr. Comp. Physiol*. 289, R45-R51. doi: 10.1152/ajpregu.00868.2004 |
| **5-HT receptor agonists** |
| Sahibzada, N., Ferreira, M., Wasserman, A.M., Taveira-DaSilva, A.M., and Gillis, RA. (2000). Reversal of morphine-induced apnea in the anesthetized rat by drugs that activate 5-hydroxytryptamine_1A_ receptors. *J. Pharmacol. Exp. Ther*. 292, 704-713.  Manzke, T., Guenther, U., Ponimaskin, E.G., Haller, M., Dutschmann M., Schwarzacher, S., and Richter, D.W. (2003). 5-HT4a receptors avert opioid-induced breathing depression without loss of analgesia. *Science* 301, 226-229. doi: 10.1126/science.1084674  Meyer, L.C., Fuller, A., and Mitchell, D. (2006). Zacopride and 8-OH-DPAT reverse opioid-induced respiratory depression and hypoxia but not catatonic immobilization in goats. *Am. J. Physiol Regul. Integr. Comp. Physiol*. 290, R405-R413. doi: 10.1152/ajpregu.00440.2005  Dutschmann, M., Waki, H., Manzke, T., Simms, A.E., Pickering, A.E., Richter, D.W., and Paton, J.F. (2009). The potency of different serotonergic agonists in counteracting opioid evoked cardiorespiratory disturbances. *Philos. Trans. R. Soc. Lond. B. Biol. Sci*. 364, 2611-2623. doi: 10.1098/rstb.2009.0076  Guenther, U., Manzke, T., Wrigge, H., Dutschmann, M., Zinserling, J., Putensen, C., and Hoeft, A. (2009). The counteraction of opioid-induced ventilatory depression by the serotonin 1A-agonist 8-OH-DPAT does not antagonize antinociception in rats *in situ* and *in vivo*. *Anesth. Analg*. 108, 1169-1176. doi: 10.1213/ane.0b013e318198f828  Manzke, T., Niebert, M., Koch, U.R., Caley, A., Vogelgesang, S., Bischoff, A.M., Hülsmann, S., Ponimaskin, E., Müller, U., Smart, T.G., Harvey, R.J., and Richter, D.W. (2011). Serotonin receptor 1A-modulated dephosphorylation of glycine receptor alpha3: a new molecular mechanism of breathing control for compensation of opioid-induced respiratory depression without loss of analgesia]. *Schmerz* 25, 272-281. doi: 10.1007/s00482-011-1044-1  Guenther, U., Theuerkauf, N.U., Huse, D., Boettcher, M.F., Wensing, G., Putensen, C., and Hoeft, A. (2012). Selective 5-HT_1A_-R-agonist repinotan prevents remifentanil-induced ventilatory depression and prolongs antinociception. *Anesthesiology* 116, 56-64. doi: 10.1097/ALN.0b013e31823d08fa  Ren, J., Ding, X., and Greer, J.J. (2015). 5-HT_1A_ receptor agonist Befiradol reduces fentanyl-induced respiratory depression, analgesia, and sedation in rats. *Anesthesiology* 122, 424-434. doi: 10.1097/ALN.0000000000000490 |
| **Glycyl-glutamine** |
| Owen, M.D., Unal, C.B., Callahan, M.F., Trivedi, K., York, C., and Millington, W.R. (2000). Glycyl-glutamine inhibits the respiratory depression, but not the antinociception, produced by morphine. *Am. J. Physiol.* 279, R1944-R1948. doi: 10.1152/ajpregu.2000.279.5.R1944 |
| **Ampakines** |
| Ren, J., Poon, B.Y, Tang Y., Funk G.D., and Greer, J.J. (2006). Ampakines alleviate respiratory depression in rats. *Am. J. Respir. Crit. Care. Med*. 174, 1384-1391. doi: 10.1164/rccm.200606-778OC  Greer, J.J., and Ren, J. (2009). Ampakine therapy to counter fentanyl-induced respiratory depression. *Respir. Physiol. Neurobiol*. 168, 153-157. doi: 10.1016/j.resp.2009.02.011.  Ren J., Ding, X., Funk, G.D., and Greer, J.J. (2009). Ampakine CX717 protects against fentanyl-induced respiratory depression and lethal apnea in rats. *Anesthesiology* 110, 1364-1370. doi: 10.1097/ALN.0b013e31819faa2a  Lorier, A.R., Funk, G.D., and Greer, J.J. (2010). Opiate-induced suppression of rat hypoglossal motoneuron activity and its reversal by ampakine therapy. *PLoS One* 5, e8766. doi: 10.1371/journal.pone.0008766  Oertel, B.G., Felden, L., Tran, P.V., Bradshaw, M.H., Angst, M.S., Schmidt, H., Johnson, S., Greer, J.J., Geisslinger, G., Varney, M.A., and Lötsch, J. (2010). Selective antagonism of opioid-induced ventilatory depression by an ampakine molecule in humans without loss of opioid analgesia. *Clin. Pharmacol. Ther*. 87, 204-211. doi: 10.1038/clpt.2009.194  Cavalla, D., Chianelli, F., Korsak, A., Hosford, P.S., Gourine, A.V., and Marina, N. (2015). Tianeptine prevents respiratory depression without affecting analgesic effect of opiates in conscious rats. *Eur. J. Pharmacol*. 761, 268-272. doi: 10.1016/j.ejphar.2015.05.067.  Haw, A.J., Meyer, L.C., Greer, J.J., Fuller, A. (2016). Ampakine CX1942 attenuates opioid-induced respiratory depression and corrects the hypoxaemic effects of etorphine in immobilized goats (Capra hircus). *Vet. Anaesth. Analg*. 43, 528-538. doi: 10.1111/vaa.12358  Dai, W., Xiao, D., Gao, X., Zhou, X.B., Fang, T.Y., Yong, Z, and Su, R.B. (2017). A brain-targeted ampakine compound protects against opioid-induced respiratory depression. *Eur. J. Pharmacol*. 809, 122-129. doi: 10.1016/j.ejphar.2017.05.025  Sun, Y., Liu, K., Martinez, E., Dale, J., Huang, D., and Wang, J. (2017). AMPAkines and morphine provide complementary analgesia. *Behav. Brain Res*. 334, 1-5. doi: 10.1016/j.bbr.2017.07.020  Dai, W., Gao, X., Xiao, D., Li, Y.L., Zhou, X.B., Yong, Z., and Su, R.B. (2019). The Impact and Mechanism of a Novel Allosteric AMPA Receptor Modulator LCX001 on Protection Against Respiratory Depression in Rodents. *Front. Pharmacol*. 10, 105. doi: 10.3389/fphar.2019.00105  Xiao, D., Xie, F., Xu, X., and Zhou, X. (2020). The impact and mechanism of ampakine CX1739 on protection against respiratory depression in rats. *Future Med. Chem*. 12, 2093-2104. doi: 10.4155/fmc-2020-0256 |
| **Micoglial inhibitor** |
| Hutchinson, M.R., Northcutt, A.L., Chao, L.W., Kearney, J.J., Zhang, Y., Berkelhammer, D.L., Loram, L.C., Rozeske, R.R., Bland, S.T., Maier, S.F., Gleeson, T.T., and Watkins, L.R. (2008). Minocycline suppresses morphine-induced respiratory depression, suppresses morphine-induced reward, and enhances systemic morphine-induced analgesia. *Brain Behav. Immun*. 22, 1248-1256. doi: 10.1016/j.bbi.2008.07.008 |
| **Thiolesters** |
| Mendoza, J., Passafaro, R., Baby, S., Young, A.P., Bates, J.N., Gaston, B., and Lewis, S.J. (2013). L-Cysteine ethyl ester reverses the deleterious effects of morphine on, arterial blood-gas chemistry in tracheotomized rats. *Respir. Physiol. Neurobiol*. 189, 136-143. doi: 10.1016/j.resp.2013.07.007  Gaston, B., Baby, S.M., May, W.J., Young, A.P., Grossfield, A., Bates, J.N., Seckler, J.M., Wilson, C.G., and Lewis, S.J. (2021). D-Cystine di(m)ethyl ester reverses the deleterious effects of morphine on ventilation and arterial blood gas chemistry while promoting antinociception. *Sci. Rep*. 11, 10038. doi: 10.1038/s41598-021-89455-2  Jenkins, M.W., Khalid, F, Baby, S.M., May, W.J., Young, A.P., Bates, J.N., Cheng, F., Seckler, J.M., and Lewis, S.J. (2021). Glutathione ethyl ester reverses the deleterious effects of fentanyl on ventilation and arterial blood-gas chemistry while prolonging fentanyl-induced analgesia. *Sci. Rep*. 11, 6985. doi: 10.1038/s41598-021-86458-x  Getsy, P.M., Baby, S.M., May, W.J., Young, A.P., Gaston, B., Hodges, M.R., Forster, H.V., Bates, J.N., Wilson, C.G., Lewis, T.H.J., Yee-Hee Hsieh, Y-H., and Stephen J. Lewis, S.J. (2022a). D-Cysteine ethyl ester reverses the deleterious effects of morphine on breathing in freely-moving rats while not negating the antinociceptive effects of the opioid. *Front. Physiol*., submitted.  Getsy, P.M., Young, A.P., Grossfield, A., Seckler, J.M., Wilson, C.G., Gaston, B., Bates, J.N., and Lewis, S.J. (2022b). D-Cysteine ethylester and D-cystine dimethylester reverse the deleterious effects of morphine on arterial blood-gas chemistry and Alveolar-arterial gradient in anesthetized rats. *Resp. Physiol. Neurobiol*., submitted. |
| **NMDA receptor antagonist** |
| Jonkman, K., van Rijnsoever, E., Olofsen, E., Aarts, L., Sarton, E., van Velzen, M., Niesters, M., and Dahan, A. (2018). Esketamine counters opioid-induced respiratory depression. *Br. J. Anaesth*. 120, 1117-1127. doi: 10.1016/j.bja.2018.02.021 |
| **Inhibition of protein kinase A and GIRK channel** |
| Liang, X., Yong, Z., and Su, R. (2018). Inhibition of protein kinase A and GIRK channel reverses fentanyl-induced respiratory depression. *Neurosci. Lett*. 677, 14-18. doi: 10.1016/j.neulet.2018.04.029 |
| **Thyrotropin Releasing Hormone and Its Analog Taltirelin** |
| Boghosian, J.D., Luethy, A., and Cotton, J.F. (2018). Intravenous and Intratracheal Thyrotropin Releasing Hormone and Its Analog Taltirelin Reverse Opioid-Induced Respiratory Depression in Isoflurane Anesthetized Rats. *J. Pharmacol. Exp. Ther*. 366, 105-112. doi: 10.1124/jpet.118.248377 |
| **Nicotinic ion-channel receptor agonists** |
| Ren, J., Ding, X., and Greer, J.J. (2019). Activating α4β2 Nicotinic Acetylcholine Receptors Alleviates Fentanyl-induced Respiratory Depression in Rats. *Anesthesiology* 130, 1017-1031. doi: 10.1097/ALN.0000000000002676  Ren, J., Ding, X., and Greer, J.J. (2020). Countering Opioid-induced Respiratory Depression in Male Rats with Nicotinic Acetylcholine Receptor Partial Agonists Varenicline and ABT 594. *Anesthesiology* 132, 1197-1211. doi: 10.1097/ALN.0000000000003128 |
| **S-nitrosothiols** |
| Getsy, P.M. Young, A.P., Gaston, B., Bates, J.N., Baby, S.M., Seckler, J.M., Grossfield, A., Jenkins, M.W., Hsieh, Y-H., and Lewis, S.J. (2022c). S-Nitroso-L-Cysteine stereoselectively blunts the negative effects of morphine on breathing and arterial blood gas chemistry while promoting analgesia. *Front. Pharmacol*., under review.  Getsy, P.M., Baby, S.M., Gruber, R.B., Gaston, B., Lewis, T.H.J., Grossfield, A., Seckler J.M, Hsieh, Y-H., Bates, J.N., and Lewis, S.J. (2022d). S-nitroso-L-cysteine stereoselectively blunts the negative effects of fentanyl on breathing while augmenting fentanyl antinociception in freely-moving rats. *Front. Pharmacol*., submitted. |

**Supplementary Table S2.** Biologically tested L-thiolester compounds

| **L-Thiolester classes** | |
| --- | --- |
| **Mono-thiolesters** | **References** |
| L-cysteine methyl ester | Wepierre et al., 1964; Shimizu et al., 1976; Yanaura et al., 1982; Krämer and Schmidt, 1984; Anderson and Holwerda, 1985; Ammon et al., 1986; Svensson, 1988a,b; Yoshikuni et al., 1988; Lailey et al. 1991, 1994; Susa et al., 1992, 1994; Ueno et al., 1992; Hobbs et al., 1993; Svensson et al., 1993; Engman et al., 1994; Fukui et al., 1994; Liu et al., 1994; Pagani et al., 1994; Bayer and Parr, 1996; Burner and Obinger, 1997; Shih et al., 1997; Ding and Demple, 1998; Burner et al., 1999; Potterf et al., 1999; Mosier-Boss and Lieberman, 2005; Manoj et al., 2006; Videhult et al., 2006; Tanaka et al., 2007; Kattaya et al., 2008; Ishii et al., 2010; Hu and Ho, 2011; Osburn et al., 2011a,b; Basu et al., 2017; Arias et al., 2019 |
| L-cysteine ethyl ester | Haas, 1965; McNiff et al., 1978; Hamilton et al., 1979; Goto et al., 1983; Krämer and Schmidt, 1984; Hisadome et al., 1986a,b, 1988, 1990; Servin et al., 1988; Svensson, 1988a; Stark et al., 1989; Schöneich et al., 1992; Susa et al., 1992, 1994; Ueno et al., 1992; Hobbs et al., 1993; Svensson et al., 1993; Fukui et al., 1994; Pagani et al., 1994; Galanakis et al., 2004; Perissinotti et al., 2005; Hu and Ho, 2011; Burner and Obinger, 1997; Ding and Demple, 1998; Burner et al., 1999; Mosier-Boss and Lieberman, 2005; Tyurina et al., 2007; Kattaya et al., 2008; Jędrzejczak et al., 2012); Defonsi Lestard et al., 2013; Mendoza et al., 2013; Arias et al., 2019 |
| L-cysteine, ethyl ester, S-(N-methylcarbamate) | Jayaram et al., 1990. |
| L-cysteine butylester | Lailey et al. 1991; Hobbs et al., 1993 |
| L-Cysteine isopropylester | Lailey et al. 1991; Butterworth et al., 1992; 1993a,b; Hobbs et al., 1993, 1998; Lailey et al., 1994. |
| L-Cysteine isobutylester | Hobbs et al., 1993 |
| L-cysteine tertiary-butylester | Hobbs et al., 1993; Lailey et al., 1994 |
| L-cysteine cyclopentylester | Hobbs et al., 1993; Lailey et al., 1994 |
| L-cysteine cyclohexylester | Butterworth et al., 1993a,b; Hobbs et al., 1993; Lailey et al., 1994 |
| L-cysteine neopentylester | Lailey et al., 1994 |
| S-(2,3-bis(palmitoyloxy)-(2-RS)-propyl)-N-palmitoyl-(R)-cysteine methyl ester | Bessler et al., 1985 |
| *N*-(*tert*-butylthiocarbamoyl)-L-cysteine ethylester | Lkhagva and Tai, 2021 |
| *N*-(*tert*-butoxycarbonyl)-l-cysteine methyl ester | Lkhagva and Tai, 2021 |
| N-(3-nitratopivaloyl)-1-cysteine-ethylester | Kojda et al., 1993, 1994 |
| N,S-dipropionyl cysteine ethyl ester | Kasumov et al., 2009 |
| γ-L-glutamylcysteine ethylester | Takimoto-Kamimura et al., 1990; Nishida et al., 1996; Henderson et al., 2016; Cauli, 2021 |
| γ-L-glutamylcysteine isopropylester | Hobbs et al., 1998 |
| L-glutathione ethylester | Levy et al., 1993; Grattagliano, et al., 1995; Minhas and Thornalley, 1995; Joshi et al., 2007; Curnow et al., 2008; Tajima et al., 2009; Nunes et al., 2011; Ito et al., 2012; Jenkins et al., 2021 |
| L-glutathione isopropylester | Karg et al., 1990; Hobbs et al., 1998 |
| L-glutathione diethylester | Levy et al., 1993 |
| L-N-acetylcysteine methyl ester | Brauer et al., 1996; Kudo et al., 1999; Bickley et al., 2004; Hay et al., 2007; Pottie et al., 2011; Poopari et al., 2015; Uesugi et al., 2016; Kularatne et al., 2020; Baby et al., 2021; Gaston et al., 2021 |
| N-acetylcysteine ethyl ester, | Kularatne et al., 2020; Tosi et al., 2021 |
| N-acetylcysteine propyl ester | Kularatne et al., 2020 |
| N-acetylcysteine butyl ester | Kularatne et al., 2020 |
| N-acetyl-S-(3-coumarinyl)-cysteine methyl ester | Eisenbrand et al., 2003 |
| N-acetyl-S-farnesyl-L-cysteine methyl ester | Tan and Rando, 1992 |
| N-acetyl-S-geranylgeranyl-L-cysteine methyl ester | Pérez-Sala et al., 1991; Tan and Rando, 1992 |
| (R)- N-acetyl-2-methylcysteine methyl ester 15 | Haya et al., 2013 |
| S-farnesyl-L-cysteine methyl ester | Liakopoulou-Kyriakides and Choli-Papadopoulou, 1992; Park et al., 1994; Baron et al., 2000 |
| S-prenylated-L-cysteine methyl ester | Kilic et al., 1997 |
| Benzoyl-glycyl-farnesyl-cysteine methyl ester | Lamango, 2005 |
| L-penicillamine ethyl ester | Horiuchi et al., 1981; Jullien et al., 2014 |
| L-penicillamine methyl ester | Karg et al., 1990; Haratake et al., 2011 |
| S-allyl-L-cysteine methylester | Herrera-R et al., 2021 |
| S-allyl-L-cysteine ethylester | Herrera-R et al., 2021 |
| S-allyl-L-Cysteine propylester | Herrera-R et al., 2021 |
| thio-2-propene-1-sulfinic acid S-allylester | Knasmüller et al., 1989; Rabinkov et al., 1998 |
| **Dithiolesters - disulfides** | |
| L-cystine diethylester | Skrede et al., 1965; Rimer et al., 2010; Laureano et al., 2016; Yang et al., 2018; Gaston et al., 2021 |
| L-cystine dimethylester | Steinherz et al., 1984; Foreman et al., 1987; Svensson, 1988; Foreman and Benson, 1990; Salmon and Baum, 1990; Coor et al., 1991; Lailey et al. 1991, 1994; Ben-Nun et al., 1992; Sakarcan et al., 1992, 1994; Chu et al., 2004; Rech et al., 2007, 2008, 2018; Figueiredo et al., 2009; Rimer et al., 2010; Sumayao et al., 2013; Sahota et al., 2014; Gurbuz et al., 2015; Yang et al., 2018; Gaston et al., 2021 |
| L-penicillamine disulfide dimethyl ester | Jullien et al., 2014 |
| L-benzyl-penicillin diethylaminoethylester | Asboe-Hansen, 1975 |
| L-glutathione diethylester | Fukumoto et al., 2010 |
| N,N-diacetyl-L-cystine dimethylester | Kitazawa et al., 2002; Chevallier et al., 2021 |
| Sulfuric acid diethylester, diethyl sulfate | Hoffman, 1980; Zhao et al., 2013 |
| Sulfuric acid dimethylester, dimethyl sulfate | Hoffman, 1980; Kisby et al., 2009 |
| Sulfuric acid dipropylester, dipropyl sulfate | Hoffman, 1980 |
| Sulfuric acid dibutylester, dibutyl sulfate | Hoffman, 1980 |

**References for Supplementary Table S2**

Ammon, H.P., Hehl, K.H., Enz, G., Setiadi-Ranti, A., and Verspohl, E.J. (1986). Cysteine analogues potentiate glucose-induced insulin release in vitro. *Diabetes* 35, 1390-1396. doi: 10.2337/diab.35.12.1390

Anderson, C.H., and Holwerda, R.A. (1985). Mechanistic flexibility in the reduction of copper(II) complexes of aliphatic polyamines by mercapto amino acids. *J. Inorg. Biochem*. 23, 29-41. doi: 10.1016/0162-0134(84)85003-5

Arias, J.M., Díaz, S.B., Ben Altabef, A., and Dupuy F.G. (2019). Interaction of cysteine and its derivatives with monolayers of dipalmitoylphosphatidylcholine. *Colloids Surf. B. Biointerfaces* 184, 110548. doi: 10.1016/j.colsurfb.2019.110548

Asboe-Hansen, G. (1975). Treatment of generalized scleroderma with inhibitors of connective tissue formation. *Acta Derm. Venereol*. 55, 461-465.

Baby, S., Gruber, R., Discala, J., Puskovic, V., Jose, N., Cheng, F., Jenkins, M., Seckler, J., and Lewis, S. (2021). Systemic Administration of Tempol Attenuates the Cardiorespiratory Depressant Effects of Fentanyl. *Front. Pharmacol*. 12, 690407. doi: 10.3389/fphar.2021.690407

Baron, R., Fourcade, E., Lajoie-Mazenc, I., Allal, C., Couderc, B., Barbaras, R., Favre, G., Faye, J.C., and Pradines, A. (2000). RhoB prenylation is driven by the three carboxyl-terminal amino acids of the protein: evidenced in vivo by an anti-farnesyl cysteine antibody. *Proc. Natl. Acad. Sci. USA*. 97, 11626-11631. doi: 10.1073/pnas.97.21.11626

Basu, A., Bhattacharjee, A., Samanta, A., and Bhattacharya, S. (2017). An oxovanadium(IV) complex protects murine bone marrow cells against cisplatin-induced myelotoxicity and DNA damage. *Drug Chem. Toxico*l. 40, 359-367. doi: 10.1080/01480545.2016.1237522

Bayer, E., and Parr, W. (1996). Elimination of hydrogen sulfide from ferredoxin and cysteine methyl ester. *Angew. Chem. Int Ed. Engl*. 5, 840-841. doi: 10.1002/anie.196608401

Ben-Nun, A., Bashan, N., Potashnik, R., Cohen-Luria, R., and Moran, A. (1992). Cystine dimethyl ester reduces the forces driving sodium-dependent transport in LLC-PK1 cells. *Am. J. Physiol*. 263, C516-C520. doi: 10.1152/ajpcell.1992.263.2.C516

Bessler, W.G., Cox, M., Lex, A., Suhr, B., Wiesmüller, K.H., and Jung, G. (1985). Synthetic lipopeptide analogs of bacterial lipoprotein are potent polyclonal activators for murine B lymphocytes. *J. Immunol*. 135, 1900-1905.

Bickley, J.F., Ciucci, A., Evans, P., Roberts, S.M., Ross, N., and Santoro, M.G. (2004). Reactions of some cyclopentenones with selected cysteine derivatives and biological activities of the product thioethers. *Bioorg. Med. Chem*. 12, 3221-3227. doi: 10.1016/j.bmc.2004.03.061

Brauer, S.L., Hneihen, A.S., McBride, J.S., and Wetterhahn, K.E. (1996). Chromium(VI) Forms Thiolate Complexes with gamma-Glutamylcysteine, N-Acetylcysteine, Cysteine, and the Methyl Ester of N-Acetylcysteine. *Inorg. Chem*. 35, 373-381. doi: 10.1021/ic941452d

Burner, U., and Obinger, C. (1997). Transient-state and steady-state kinetics of the oxidation of aliphatic and aromatic thiols by horseradish peroxidase. *FEBS Lett*. 411, 269-274. doi: 10.1016/s0014-5793(97)00713-8

Burner, U., Jantschko, W., and Obinger, C. (1999). Kinetics of oxidation of aliphatic and aromatic thiols by myeloperoxidase compounds I and II. *FEBS Lett*. 443, 290-296. doi: 10.1016/s0014-5793(98)01727-x

Butterworth, M., Upshall, D.G., Smith, L.L., and Cohen, G.M. (1992). Cysteine isopropylester protects against paracetamol-induced toxicity. *Biochem. Pharmacol*. 43, 483-488. doi: 10.1016/0006-2952(92)90567-3

Butterworth, M., Upshall, D.G., and Cohen, G.M. (1993a). A novel role for carboxylesterase in the elevation of cellular cysteine by esters of cysteine. *Biochem. Pharmacol*. 46, 1131-1137. doi: 10.1016/0006-2952(93)90460-e

Butterworth, M., Upshall, D.G., Hobbs, M., and Cohen, G.M. (1993b). Elevation of cysteine and replenishment of glutathione in rat lung slices by cysteine isopropylester and other cysteine precursors. *Biochem. Pharmacol*. 45, 1769-1774. doi: 10.1016/0006-2952(93)90432-v

Cauli, O. (2021). Oxidative Stress and Cognitive Alterations Induced by Cancer Chemotherapy Drugs: A Scoping Review. *Antioxidants (Basel)* 10, 1116. doi: 10.3390/antiox10071116

Chevallier, V., Zoller, M., Kochanowski, N., Andersen, M.R., Workman, C.T., and Malphettes, L. (2021). Use of novel cystine analogs to decrease oxidative stress and control product quality. *J. Biotechnol*. 327, 1-8. doi: 10.1016/j.jbiotec.2020.12.011

Chu, F., Chen, L.H., and O'Brian, C.A. (2004). Cellular protein kinase C isozyme regulation by exogenously delivered physiological disulfides - implications of oxidative protein kinase C regulation to cancer prevention. *Carcinogenesis* 25, 585-596. doi: 10.1093/carcin/bgh041

Coor, C., Salmon, R.F., Quigley, R., Marver, D., and Baum, M. (1991). Role of adenosine triphosphate (ATP) and NaK ATPase in the inhibition of proximal tubule transport with intracellular cystine loading. *J. Clin. Invest*. 87, 955-961. doi: 10.1172/JCI115103

Curnow, E.C., Ryan, J., Saunders, D., and Hayes, E.S. (2008). Bovine in vitro oocyte maturation as a model for manipulation of the gamma-glutamyl cycle and intraoocyte glutathione. *Reprod. Fertil. Dev*. 20, 579-588. doi: 10.1071/rd08041

Defonsi Lestard, M.E., Díaz, S.B., Puiatti, M., Echeverría, G.A., Piro, O.E., Pierini, A.B., Altabef, A.B., and Tuttolomondo, M.E. (2013). Vibrational and structural behavior of (L)-cysteine ethyl ester hydrochloride in the solid state and in aqueous solution. *J. Phys. Chem. A*. 117, 14243-14252. doi: 10.1021/jp409252d

Ding, H., and Demple, B. (1998). Thiol-mediated disassembly and reassembly of [2Fe-2S] clusters in the redox-regulated transcription factor SoxR. *Biochemistry* 37, 17280-17286. doi: 10.1021/bi980532g

Eisenbrand, G., Otteneder, M., and Tang, W. (2003). Synthesis of N-acetyl-S-(3-coumarinyl)-cysteine methyl ester and HPLC analysis of urinary coumarin metabolites. *Toxicology* 190, 249-258. doi: 10.1016/s0300-483x(03)00204-x

Engman, L., Tunek, A., Hallberg, M., and Hallberg, A. (1994). Catalytic effects of glutathione peroxidase mimetics on the thiol reduction of cytochrome c. *Chem. Biol. Interact*. 93, 129-137. doi: 10.1016/0009-2797(94)90092-2

Figueiredo, V.C., Feksa L.R., and Wannmacher C.M. (2009). Cysteamine prevents inhibition of adenylate kinase caused by cystine in rat brain cortex. *Metab. Brain Dis*. 24, 723-731. doi: 10.1007/s11011-009-9141-x

Foreman, J.W., Bowring, M.A., Lee, J., States, B., and Segal, S. (1987). Effect of cystine dimethylester on renal solute handling and isolated renal tubule transport in the rat: a new model of the Fanconi syndrome. *Metabolism* 36, 1185-1191. doi: 10.1016/0026-0495(87)90246-0

Foreman, J.W., and Benson, L. (1990). Effect of cystine loading and cystine dimethylester on renal brush border membrane transport. *Biosci. Rep*. 10, 455-459. doi: 10.1007/BF01152292

Fukui, K., Kaneda, M., Takahashi, E., Washio, M., and Doi, K. (1994). Protective effects of sulfhydryl compounds on HOCl-induced intracellular Ca^2+^ increase in single rat ventricular myocytes. *J. Mol. Cell Cardiol*. 26, 455-461. doi: 10.1006/jmcc.1994.1056

Fukumoto, A., Maruyama, K., Walsh, T., Kajiya, K., Hamuro, J., D'Amore, P.A., and Kinoshita, S. (2010a). Intracellular thiol redox status regulates lymphangiogenesis and dictates corneal limbal graft survival. *Invest. Ophthalmol. Vis. Sci*. 51, 2450-2458. doi: 10.1167/iovs.09-4618

Galanakis, D., Kourounakis, A.P., Tsiakitzis, K.C., Doulgkeris, C., Rekka, E.A., Gavalas, A, Kravaritou, C., Charitos, C., and Kourounakis, P.N. (2004). Synthesis and pharmacological evaluation of amide conjugates of NSAIDs with L-cysteine ethyl ester, combining potent antiinflammatory and antioxidant properties with significantly reduced gastrointestinal toxicity. *Bioorg. Med. Chem. Lett*. 14, 3639-3643. doi: 10.1016/j.bmcl.2004.05.025

Gaston, B., Baby, S.M., May, W.J., Young, A.P., Grossfield, A., Bates, J.N., Seckler, J.M., Wilson, C.G., and Lewis, S.J. (2021). D-Cystine di(m)ethyl ester reverses the deleterious effects of morphine on ventilation and arterial blood gas chemistry while promoting antinociception. *Sci. Rep*. 11, 10038. doi: 10.1038/s41598-021-89455-2

Goto, K., Hisadome, M., Kawazoe, Y., and Tsumagari, T. (1983). Effect of cysteine ethylester hydrochloride (Cystanin) on host defense mechanism]. *Nihon Yakurigaku Zasshi* 82, 27-35.

Grattagliano, I., Wieland, P., Schranz, C., and Lauterburg, B.H. (1995). Disposition of glutathione monoethyl ester in the rat: glutathione ester is a slow release form of extracellular glutathione. *J. Pharmacol. Exp. Ther*. 272, 484-488.

Gurbuz, N., Park, M.A., Dent, P., Abdel Mageed, A.B., Sikka, S.C., and Baykal, A. (2015). Cystine Dimethyl Ester Induces Apoptosis Through The Regulation of PKC-δ and PKC-ε in Prostate Cancer Cells. *Anticancer Agents Med. Chem*. 15, 217-227. doi: 10.2174/1871520614666141120121901

Haratake, M., Sakano, T., Fuchigami, T., and Nakayama, M. (2011). Thiol-targeted introduction of selenocysteine to polypeptides for synthesis of glutathione peroxidase mimics. *Metallomics* 3, 702-709. doi: 10.1039/c1mt00001b

Haas, D.J. (1965). Intermolecular complexes. 3. l-Cysteine ethyl ester hydrochloride-urea (1:1). *Acta Crystallogr*. 19, 860-861. doi: 10.1107/s0365110x65004498

Hamilton, G.A., Buckthal, D.J., Mortensen, R.M., and Zerby, K.W. (1979). Reactions of cysteamine and other amine metabolites with glyoxylate and oxygen catalyzed by mammalian D-amino acid oxidase. *Proc. Natl. Acad. Sci. USA*. 76, 2625-2629. doi: 10.1073/pnas.76.6.2625

Hay, S., Westerlund, K., and Tommos, C. (2007). Redox characteristics of a de novo quinone protein. *J. Phys. Chem. B*. 111, 3488-3495. doi: 10.1021/jp066654x

Haya, L., Osante, I., Mainar, A.M., Cativiela, C., and Urieta, J.S. (2013). *Phys. Chem. Chem. Phys*. 15, 9407-13. doi: 10.1039/c3cp50743b

Henderson, M., Rice, B., Sebastian, A., Sullivan, P.G., King, C., Robinson, R.A., and Reed T.T. (2016). Neuroproteomic study of nitrated proteins in moderate traumatic brain injured rats treated with gamma glutamyl cysteine ethyl ester administration post injury: Insight into the role of glutathione elevation in nitrosative stress. *Proteomics Clin. Appl*. 10, 1218-1224. doi: 10.1002/prca.201600004

Herrera-R, A., Castrillón, W., Pastrana, M., Yepes, A.F., and Cardona-G, W. (2021). Promising Hybrids Derived from S-Allylcysteine and NSAIDs Fragments against Colorectal Cancer: Synthesis, In-vitro Evaluation, Drug-Likeness and In-silico ADME/tox Studies. *Iran J. Pharm. Res*. 20, 351-367. doi: 10.22037/ijpr.2020.114347.14806

Hisadome, M., Nakamura, Y., Okumoto, T., and Ikegami, K. (1986a). Effect of cysteine ethylester hydrochloride (Cystanin) on host defense mechanisms (II): Restorative effects on the suppression of antibody production. *Nihon Yakurigaku Zasshi* 88, 349-354. doi: 10.1254/fpj.88.349

Hisadome, M., Nakamura, Y., Okumoto, T., and Ikegami, K. (1986b). Effect of cysteine ethylester hydrochloride (Cystanin) on host defense mechanisms (III): Potentiating effects on phagocytosis and nitroblue tetrazolium (NBT) reduction by leukocytes of mice and guinea pigs. *Nihon Yakurigaku Zasshi*. 88, 369-374. doi: 10.1254/fpj.88.369

Hisadome, M., Kimura, Y., Ikegami, K., Terasawa, M. (1988). Effect of cysteine ethylester hydrochloride (Cystanin) on host defense mechanism (IV): Potentiating effects on the function of peritoneal or spleen macrophages. *Jpn. J. Pharmacol*. 47, 379-385. doi: 10.1254/jjp.47.379

Hisadome, M., Fukuda, T., and Terasawa, M. (1990). Effect of cysteine ethylester hydrochloride (Cystanin) on host defense mechanisms (V): Potentiation of nitroblue tetrazolium reduction and chemiluminescence in macrophages or leukocytes of mice or rats. *Jpn. J. Pharmacol*. 53, 57-66. doi: 10.1254/jjp.53.57

Hobbs, M.J., Butterworth, M., Cohen, G.M., and Upshall D.G. (1993). Structure-activity relationships of cysteine esters and their effects on thiol levels in rat lung in vitro. *Biochem. Pharmacol*. 45, 1605-1612. doi: 10.1016/0006-2952(93)90301-c

Hobbs, M.J., Williams, N.E., Patel, S.K., and Upshall, D.G. (1998). Elevation of endogenous nucleophiles in rat lung by cysteine and glutathione esters in vitro. *Biochem. Pharmacol*. 55, 1573-1584. doi: 10.1016/s0006-2952(97)00674-6

Hoffmann, G.R. (1980). Genetic effects of dimethyl sulfate, diethyl sulfate, and related compounds. *Mutat. Res*. 75, 63-129. doi: 10.1016/0165-1110(80)90028-7

Horiuchi, K., Yokoyama, A., Tsuiki, K., Tanaka, H., and Saji, H. (1981). Effect of the nature of technetium coordination complex on cell membrane permeability: mononuclear complex of 99mTc-penicillamine ethyl ester. Int. *J. Appl. Radiat. Isot*. 32, 545-351. doi: 10.1016/0020-708x(81)90032-6

Hu, T.M., and Ho, S.C. (2011). Similarity and dissimilarity of thiols as anti-nitrosative agents in the nitric oxide-superoxide system. *Biochem. Biophys. Res. Commun*. 404, 785-789. doi: 10.1016/j.bbrc.2010.12.059

Ishii, T., Wakabayashi, M., Mori, T., and Nakayama, T. (2010). A new method for the detection and characterization of alpha-lipoic acid mixed disulphides. *Free Radic. Res*. 44, 403-409. doi: 10.3109/10715760903536331

Ito, L., Okumura, M., Tao, K., Kasai, Y., Tomita, S., Oosuka, A., Yamada, H., Shibano, T., Shiraki, K., Kumasaka, T., and Yamaguchi, H. (2012). Glutathione ethylester, a novel protein refolding reagent, enhances both the efficiency of refolding and correct disulfide formation. *Protein J*. 31, 499-503. doi: 10.1007/s10930-012-9427-4

Jayaram, H.N., Lui, M.S., Plowman, J., Pillwein, K., Reardon, M.A., Elliott, W.L., and Weber, G. (1990). Oncolytic activity and mechanism of action of a novel L-cysteine derivative, L-cysteine, ethyl ester, S-(N-methylcarbamate) monohydrochloride. *Cancer Chemother. Pharmacol*. 26, 88-92. doi: 10.1007/BF02897250

Jędrzejczak, R., Wojciechowski, M., Andruszkiewicz, R., Sowiński, P., Kot-Wasik, A., and Milewski, S. (2012). Inactivation of glucosamine-6-phosphate synthase by N3-oxoacyl derivatives of L-2,3-diaminopropanoic acid. *Chembiochem*. 13, 85-96. doi: 10.1002/cbic.201100587

Jenkins, M.W., Khalid, F, Baby, S.M., May, W.J., Young, A.P., Bates, J.N., Cheng, F., Seckler, J.M., and Lewis, S.J. (2021). Glutathione ethyl ester reverses the deleterious effects of fentanyl on ventilation and arterial blood-gas chemistry while prolonging fentanyl-induced analgesia. *Sci. Rep*. 11, 6985. doi: 10.1038/s41598-021-86458-x

Joshi, G, Hardas, S., Sultana, R., St Clair, D.K., Vore, M., and Butterfield, D.A. (2007). Glutathione elevation by gamma-glutamyl cysteine ethyl ester as a potential therapeutic strategy for preventing oxidative stress in brain mediated by in vivo administration of adriamycin: Implication for chemobrain. *J. Neurosci. Res*. 85, 497-503. doi: 10.1002/jnr.21158

Jullien, A.S., Gateau, C., Lebrun, C., Kieffer, I., Testemale, D., and Delangle, P. (2014). D-Penicillamine tripodal derivatives as efficient copper(I) chelators. *Inorg. Chem*. 53, 5229-5239. doi: 10.1021/ic5004319

Karg, E., Tunek, A., Brötell, H., Hallberg, A., Rosengren, E., and Rorsman, H. (1990). Glutathione in human melanoma cells. Effects of cysteine, cysteine esters and glutathione isopropyl ester. *J. Dermatol. Sci*. 1, 39-45. doi: 10.1016/0923-1811(90)90008-2

Kasumov, T., Sharma, N., Huang, H., Kombu, R.S., Cendrowski, A., Stanley, W.C., and Brunengraber, H. (2009). Dipropionylcysteine ethyl ester compensates for loss of citric acid cycle intermediates during post ischemia reperfusion in the pig heart. *Cardiovasc. Drugs Ther*. 23, 459-69. doi: 10.1007/s10557-009-6208-1

Kattaya, S.A., Akkus, O., and Slama, J. (2008). Radioprotectant and radiosensitizer effects on sterility of gamma-irradiated bone. *Clin. Orthop. Relat. Res*. 466, 1796-1803. doi: 10.1007/s11999-008-0283-7

Kilic, F., Dalton, M.B., Burrell, S.K., Mayer, J.P., Patterson, S.D., and Sinensky, M. (1997). In vitro assay and characterization of the farnesylation-dependent prelamin A endoprotease. *J. Biol. Chem*. 272, 5298-304. doi: 10.1074/jbc.272.8.5298

Kisby, G.E., Olivas, A., Park, T., Churchwell, M., Doerge, D., Samson, L.D., Gerson, S.L., and Turker, M.S. (2009). DNA repair modulates the vulnerability of the developing brain to alkylating agents. *DNA Repair. (Amst)*. 8, 400-412. doi: 10.1016/j.dnarep.2008.12.002

Kitazawa, M., Nakano, T., Chuujou, H., Shiojiri, E., Iwasaki, K., and Sakamoto, K. (2002). Intracellular redox regulation by a cystine derivative suppresses UV-induced NF-kappa B activation. *FEBS Lett*. 526, 106-110. doi: 10.1016/s0014-5793(02)03152-6

Knasmüller, S., de Martin, R., Domjan, G., and Szakmary, A. (1989). Studies on the antimutagenic activities of garlic extract. *Environ. Mol. Mutagen*. 13, 357-365. doi: 10.1002/em.2850130413

Kojda, G., Meyer, W., and Noack, E. (1993). Influence of endothelium and nitrovasodilators on free thiols and disulfides in porcine coronary smooth muscle. *Eur. J. Pharmacol*. 250, 385-394. doi: 10.1016/0014-2999(93)90025-d

Kojda, G., Beck, J.K., Meyer, W., and Noack, E. (1994). Nitrovasodilator-induced relaxation and tolerance development in porcine vena cordis magna: dependence on intact endothelium. *Br. J. Pharmacol*. 112, 533-540. doi: 10.1111/j.1476-5381.1994.tb13106.x

Krämer, E, and Schmidt, A. (1984). Oxidation of cysteine to cystine by membrane fractions of Chlorella fusca. *Planta* 160, 235-241. doi: 10.1007/BF00402860

Kudo, N., Matsumori, N., Taoka, H., Fujiwara, D., Schreiner, E.P., Wolff, B., Yoshida, M., and Horinouchi, S. (1999). Leptomycin B inactivates CRM1/exportin 1 by covalent modification at a cysteine residue in the central conserved region. *Proc. Natl. Acad. Sci. USA*. 96, 9112-9117. doi: 10.1073/pnas.96.16.9112

Kularatne, R.N., Bulumulla, C., Catchpole, T., Takacs, A., Christie, A., Stefan, M.C., and Csaky, K.G. (2020). Protection of human retinal pigment epithelial cells from oxidative damage using cysteine prodrugs. *Free Radic. Biol. Med*. 152, 386-394. doi: 10.1016/j.freeradbiomed.2020.03.024

Lailey, A.F., Hill, L., Lawston, I.W., Stanton, D., and Upshall, D.G. (1991). Protection by cysteine esters against chemically induced pulmonary oedema. *Biochem. Pharmacol*. 42 Suppl, S47-S54. doi: 10.1016/0006-2952(91)90391-h

Lailey, A.F., and Upshall, D.G. (1994). Thiol levels in rat bronchio-alveolar lavage fluid after administration of cysteine esters. *Hum. Exp. Toxicol*. 13, 776-780. doi: 10.1177/096032719401301106

Lamango, N.S. (2005). Liver prenylated methylated protein methyl esterase is an organophosphate-sensitive enzyme. J. Biochem. Mol. Toxicol. 19, 347-57. doi: 10.1002/jbt.20100

Laureano, M.R., Onishi, E.T., Bressan, R.A., Neto, P.B., Castiglioni, M.L., Batista, I.R, Reis, M.A., Garcia, M.V., de Andrade, A.N., Sanchez, M.L., Moreira, H.C., de Almeida, R.R., Garrido, G.J., and Jackowski, A.P. (2016). The effectiveness of acupuncture as a treatment for tinnitus: a randomized controlled trial using (99m)Tc-ECD SPECT. *Eur. Radiol*. 26, 3234-3242. doi: 10.1007/s00330-015-4164-7

Levy, E.J., Anderson, M.E., and Meister, A. (1993). Transport of glutathione diethyl ester into human cells. *Proc. Natl. Acad. Sci. USA*. 90, 9171-9175. doi: 10.1073/pnas.90.19.9171

Liakopoulou-Kyriakides, M., and Choli-Papadopoulou, T. (1992). Synthesis of S-farnesyl-L-cysteine methylester and purification by HPLC. *Amino Acids* 2, 285-288. doi: 10.1007/BF00805949

Liu, X., Gillespie, J.S., and Martin, W. (1994). Non-adrenergic, non-cholinergic relaxation of the bovine retractor penis muscle: role of S-nitrosothiols. *Br. J. Pharmacol*. 111, 1287-1295. doi: 10.1111/j.1476-5381.1994.tb14885.x

Lkhagva, A., and Tai, H.C. (2021). Dimethylcysteine (DiCys)/o-Phthalaldehyde Derivatization for Chiral Metabolite Analyses: Cross-Comparison of Six Chiral Thiols. *Molecules* 26, 7416. doi: 10.3390/molecules26247416

Manoj, V.M., Mohan, H., Aravind, U.K., and Aravindakumar, C.T. (2006). One-electron reduction of S-nitrosothiols in aqueous medium. *Free Radic. Biol. Med*. 41, 1240-1246. doi: 10.1016/j.freeradbiomed.2006.06.025

McNiff, E.F., Cheng, L.K., Woodfield, H.C., and Fung, H.L. (1978). Effects of L-cysteine, L-cysteine derivatives and ascorbic acid on lead excretion in rats. *Res. Commun. Chem. Pathol. Pharmacol*. 20, 131-137.

Mendoza, J., Passafaro, R., Baby, S., Young, A.P., Bates, J.N., Gaston, B., and Lewis, S.J. (2013). L-Cysteine ethyl ester reverses the deleterious effects of morphine on, arterial blood-gas chemistry in tracheotomized rats. *Respir. Physiol. Neurobiol*. 189, 136-143. doi: 10.1016/j.resp.2013.07.007

Minhas, H.S., and Thornalley, P.J. (1995). Comparison of the delivery of reduced glutathione into P388D1 cells by reduced glutathione and its mono- and diethyl ester derivatives. *Biochem. Pharmacol*. 49, 1475-1482. doi: 10.1016/0006-2952(94)00518-q

Mosier-Boss, P.A., and Lieberman, S.H. (2005). The role of hydrogen bonding in the selectivity of L-cysteine methyl ester (CYSM) and L-cysteine ethyl ester (CYSE) for chloride ion. *Spectrochim. Acta A. Mol. Biomol. Spectrosc*. 61, 845-54. doi: 10.1016/j.saa.2004.06.011

Nishida, K., Ohta, Y., Ito, M., Nagamura, Y., Kitahara, S., Fujii, K., and Ishiguro, I. (1996) Conversion of gamma-glutamylcysteinylethyl ester to glutathione in rat hepatocytes. *Biochim. Biophys. Acta*. 1313, 47-53. doi: 10.1016/0167-4889(96)00054-7

Osburn, S., Steill, J.D., Oomens, J., O'Hair, R.A., van Stipdonk, M., and Ryzhov, V. (2011a). Structure and reactivity of the cysteine methyl ester radical cation. *Chemistry* 17, 873-879. doi: 10.1002/chem.201002042

Osburn, S., O'Hair, R.A., Black, S.M., and Ryzhov, V. (2011b). Post-translational modification in the gas phase: mechanism of cysteine S-nitrosylation via ion-molecule reactions. *Rapid Commun. Mass Spectrom*. 25, 3216-3222. doi: 10.1002/rcm.5219

Pagani, R., Leoncini, R., Terzuoli, L., Pizzichini, M., and Marinello, E. (1994). The regulation of alanine and aspartate aminotransferase by different aminothiols and by vitamin B-6 derivatives. *Biochim. Biophys. Acta*. 1204, 250-256. doi: 10.1016/0167-4838(94)90015-9

Park, S.B., Howald, W.N., and Cashman, J.R. (1994). S-oxidative cleavage of farnesylcysteine and farnesylcysteine methyl ester by the flavin-containing monooxygenase. *Chem. Res. Toxicol*. 7, 191-198. doi: 10.1021/tx00038a012

Pérez-Sala, D., Tan, E.W., Cañada, F.J., and Rando, R.R. (1991). Methylation and demethylation reactions of guanine nucleotide-binding proteins of retinal rod outer segments. *Proc. Natl. Acad. Sci. USA*. 88, 3043-3046. doi: 10.1073/pnas.88.8.3043

Perissinotti, L.L., Turjanski, A.G., Estrin, D.A., and Doctorovich, F. (2005). Transnitrosation of nitrosothiols: characterization of an elusive intermediate. *J. Am. Chem. Soc*. 127, 486-487. doi: 10.1021/ja044056v

Poopari, M.R., Dezhahang, Z., and Xu, Y. (2015). Identifying dominant conformations of N-acetyl-L-cysteine methyl ester and N-acetyl-L-cysteine in water: VCD signatures of the amide I and the C=O stretching bands. *Spectrochim. Acta A. Mol. Biomol. Spectrosc*. 136, 131-140. doi: 10.1016/j.saa.2013.08.118

Potterf, S.B., Virador, V., Wakamatsu, K., Furumura, M., Santis, C., Ito, S., and Hearing V.J. (1999). Cysteine transport in melanosomes from murine melanocytes. *Pigment Cell Res*. 12, 4-12. doi: 10.1111/j.1600-0749.1999.tb00502.x

Pottie, I.R., Higgins, E.A., Blackman, R.A., Macdonald, I.R., Martin, E., and Darvesh S. (2011). Cysteine thioesters as myelin proteolipid protein analogues to examine the role of butyrylcholinesterase in myelin decompaction. *ACS Chem. Neurosci*. 2, 151-159. doi: 10.1021/cn100090g

Rabinkov, A., Miron, T., Konstantinovski, L., Wilchek, M., Mirelman, D., and Weiner, L. (1998). The mode of action of allicin: trapping of radicals and interaction with thiol containing proteins. *Biochim. Biophys. Acta* 1379, 233-244. doi: 10.1016/s0304-4165(97)00104-9

Rech, V.C., Feksa, L.R., Arevalo do Amaral, M.F., Koch, G.W., Wajner, M., Dutra-Filho, C.S., Terezinha de Souza Wyse, A., and Duval Wannmacher, C.M. (2007). Promotion of oxidative stress in kidney of rats loaded with cystine dimethyl ester. *Pediatr. Nephrol*. 22, 1121-1128. doi: 10.1007/s00467-007-0494-2

Rech, V.C., Feksa, L.R., Fleck, R.M., Athaydes, G.A., Dornelles, P.K., Rodrigues-Junior, V., and Wannmacher, C.M. (2008). Cysteamine prevents inhibition of thiol-containing enzymes caused by cystine or cystine dimethylester loading in rat brain cortex. *Metab. Brain Dis*. 23, 133-145. doi: 10.1007/s11011-008-9081-x

Rech, V.C., Mezzomo, N.J., Athaydes, G.A., Feksa, L.R., Figueiredo, V.C., Kessler, A., Franceschi, I.D., and Wannmacher, C.M.D. (2018). Thiol/disulfide status regulates the activity of thiol-containing kinases related to energy homeostasis in rat kidney. *An. Acad. Bras. Cienc*. 90, 99-108. doi: 10.1590/0001-3765201720160348

Rimer, J.D., An, Z., Zhu Z., Lee, M.H., Goldfarb, D.S., Wesson, J.A., and Ward, M.D. (2010). Crystal growth inhibitors for the prevention of L-cystine kidney stones through molecular design. *Science* 330, 337-341. doi: 10.1126/science.1191968

Sahota, A., Parihar, J.S., Capaccione, K.M., Yang, M., Noll, K., Gordon, D., Reimer D., Yang, I., Buckley, B.T., Polunas, M., Reuhl, K.R., Lewis, M.R., Ward, M.D., Goldfarb, D.S., and Tischfield, J.A. (2014). Novel cystine ester mimics for the treatment of cystinuria-induced urolithiasis in a knockout mouse model. *Urology* 84, 1249, e9-e15. doi: 10.1016/j.urology.2014.07.043

Sakarcan, A., Aricheta, R., and Baum, M. (1992). Intracellular cystine loading causes proximal tubule respiratory dysfunction: effect of glycine. *Pediatr. Res*. 32, 710-713. doi: 10.1203/00006450-199212000-00018

Sakarcan, A., Timmons, C., and Baum, M. (1994). Intracellular distribution of cystine in cystine-loaded proximal tubules. *Pediatr. Res*. 35, 447-50

Salmon, R.F., and Baum, M. (1990). Intracellular cystine loading inhibits transport in the rabbit proximal convoluted tubule. *J. Clin. Invest*. 85, 340-344. doi: 10.1172/JCI114443. PMID: 2298908

Schöneich, C., Dillinger, U., von Bruchhausen, F., and Asmus, K.D. (1992). Oxidation of polyunsaturated fatty acids and lipids through thiyl and sulfonyl radicals: reaction kinetics, and influence of oxygen and structure of thiyl radicals. *Arch. Biochem. Biophys*. 292, 456-467. doi: 10.1016/0003-9861(92)90016-p

Servin, A.L., Goulinet, S., and Renault, H. (1988). Pharmacokinetics of cysteine ethyl ester in rat. *Xenobiotica* 18, 839-847. doi: 10.3109/00498258809041722

Shih, T.W., Lin T.H., Shealy Y.F., and Hill, DL. (1997). Nonenzymatic isomerization of 9-cis-retinoic acid catalyzed by sulfhydryl compounds. *Drug Metab. Dispos*. 25, 27-32.

Shimizu, T., Nozawa, T., and Hatano, M. (1976). Magnetic circular dichroism of myoglobin-thiolate complexes. *Biochim. Biophys. Acta*. 434, 126-36. doi: 10.1016/0005-2795(76)90042-8

Skrede, S., Bremer, J., and Eldjarn, L. (1965). The Effect of Disulphides on Mitochondrial Oxidations. *Biochem. J*. 95, 838-846.

Stark, A.A., Arad, A., Siskindovich, S., Pagano, D.A., and Zeiger, E. (1989). Effect of pH on mutagenesis by thiols in Salmonella typhimurium TA102. *Mutat. Res*. 224, 89-94. doi: 10.1016/0165-1218(89)90007-4

Steinherz, R., Makov, N., Narinsky, R., Meidan, B., and Kohn, G. (1984). Comparative study of cystine clearance in cystinotic and I-cell fibroblasts upon exposure to cystine dimethyl ester. *Enzyme* 32, 126-130. doi: 10.1159/000469462

Sumayao, R., McEvoy, B., Martin-Martin, N., McMorrow, T., and Newsholme, P. (2013). Cystine dimethylester loading promotes oxidative stress and a reduction in ATP independent of lysosomal cystine accumulation in a human proximal tubular epithelial cell line. *Exp. Physiol*. 98, 1505-15017. doi: 10.1113/expphysiol.2013.073809

Susa, N., Ueno, S., and Furukawa, Y. (1992). Protective effects of thiol compounds on chromate-induced cytotoxicity in HeLa cells. *J. Vet. Med. Sci*. 54, 281-288. doi: 10.1292/jvms.54.281

Susa, N., Ueno, S., and Furukawa, Y. (1994). Protective effects of thiol compounds on chromate-induced toxicity *in vitro* and *in vivo*. *Environ. Health Perspect*. 102 (Suppl 3), 247-250. doi: 10.1289/ehp.94102s3247

Svensson, B.E. (1988a). Abilities of peroxidases to catalyse peroxidase-oxidase oxidation of thiols. *Biochem. J*. 256, 757-762. doi: 10.1042/bj2560757

Svensson, B.E. (1988b). Thiols as myeloperoxidase-oxidase substrates. *Biochem. J*. 253, 441-449. doi: 10.1042/bj2530441

Svensson, B.E., Gräslund, A., Ström, G., and Moldeus, P. (1993). Thiols as peroxidase substrates. *Free Radic. Biol. Med*. 14, 167-175. doi: 10.1016/0891-5849(93)90007-h

Tajima, M., Kurashima, Y., Sugiyama, K., Ogura, T., and Sakagami, H. (2009). The redox state of glutathione regulates the hypoxic induction of HIF-1. *Eur. J. Pharmacol*. 606, 45-49. doi: 10.1016/j.ejphar.2009.01.026

Takimoto-Kamimura, M., Koyano, K., Kitahara, S., and Fujii, K. (1990). Structure of N-gamma-L-glutamyl-L-cysteine ethyl ester monohydrate. *Acta Crystallogr. C*. 46, 2247-9. doi: 10.1107/s0108270190000774

Tan, E.W., and Rando R.R. (1992). Identification of an isoprenylated cysteine methyl ester hydrolase activity in bovine rod outer segment membranes. *Biochemistry* 31, 5572-5578. doi: 10.1021/bi00139a021

Tanaka, T., Nakashima, T., Ueda, T., Tomii, K., and Kouno, I. (2007). Facile discrimination of aldose enantiomers by reversed-phase HPLC. Chem. Pharm. Bull. (Tokyo). 55, 899-901. doi: 10.1248/cpb.55.899

Tosi, G.M., Giustarini, D., Franci, L., Minetti, A., Imperatore, F., Caldi, E., Fiorenzani, P., Aloisi, A.M., Sparatore, A., Rossi, R., Chiariello, M., Orlandini, M., and Galvagni, F. (2021). Superior Properties of N-Acetylcysteine Ethyl Ester over N-Acetyl Cysteine to Prevent Retinal Pigment Epithelial Cells Oxidative Damage. *Int. J. Mol. Sci*. 22, 600. doi: 10.3390/ijms22020600

Tyurina, Y.Y., Basova, L.V., Konduru, N.V., Tyurin, V.A., Potapovich, A.I., Cai, P., Bayir, H., Stoyanovsky, D., Pitt, B.R., Shvedova, A.A., Fadeel, B., and Kagan, V.E. (2007). Nitrosative stress inhibits the aminophospholipid translocase resulting in phosphatidylserine externalization and macrophage engulfment: implications for the resolution of inflammation. *J. Biol. Chem*. 282, 8498-8509. doi: 10.1074/jbc.M606950200

Ueno, S. (1992). Protective effects of thiol containing chelating agents against liver injury induced by hexavalent chromium in mice. *Kitasato Arch. Exp. Med*. 65, 87-96.

Uesugi, S., Fujisawa, N., Yoshida, J., Watanabe, M., Dan, S., Yamori, T., Shiono, Y., and Kimura, K. (2016). Pyrrocidine A, a metabolite of endophytic fungi, has a potent apoptosis-inducing activity against HL60 cells through caspase activation via the Michael addition. *J. Antibiot. (Tokyo)*. 69, 133-140. doi: 10.1038/ja.2015.103

Videhult, P., Laurell, G., Wallin, I., and Ehrsson, H. (2006). Kinetics of Cisplatin and its monohydrated complex with sulfur-containing compounds designed for local otoprotective administration. *Exp. Biol. Med. (Maywood)*. 231, 1638-45. doi: 10.1177/153537020623101009

Wepierre, J., Laprand, J., Garcet, S., and Laurre, M. (1964). Etude de la repartition et de l’elimination de l’ester methylique de la cysteine ^35^S chez la Souris et le Rat. *Therapie* 19, 187-200

Yanaura, S., Takeda, H., and Misawa, M. (1982). Behavior of mucus glycoproteins of tracheal secretory cells following L-cysteine methyl ester treatment. *J. Pharmacobiodyn*. 5, 603-610. doi: 10.1248/bpb1978.5.603

Yang, Y., Albanyan, H., Lee, S., Aloysius, H., Liang, J.J., Kholodovych, V., Sahota, A., and Hu, L. (2018). Design, synthesis, and evaluation of l-cystine diamides as l-cystine crystallization inhibitors for cystinuria. Bioorg. *Med. Chem. Lett*. 28, 1303-1308. doi: 10.1016/j.bmcl.2018.03.024

Yoshikuni, M., Ishikawa, K., Isobe, M., Goto, T., and Nagahama, Y. (1988). Synergistic actions of disulfide-reducing agents on 1-methyladenine-induced oocyte maturation in starfish. *Dev. Biol*. 128, 236-239. doi: 10.1016/0012-1606(88)90285-0

Zhao, P., Fu, J., Yao, B, Hu, E., Song, Y., Mi, L., Li, Z., Zhang, H., Jia, Y., Ma, S., Chen, W., and Zhou, Z. (2013). Diethyl sulfate-induced cell cycle arrest and apoptosis in human bronchial epithelial 16HBE cells. *Chem. Biol. Interact*. 205, 81-89. doi: 10.1016/j.cbi.2013.06.014

**Supplementary Table S3.** Definition of ventilatory parameters recorded

| **Parameter** | **Abbreviation** | | **Units** | **Definition** |
| --- | --- | --- | --- | --- |
| **A. Directly recorded parameters** | | | | |
| Frequency of breaths | Freq | | breaths/min | Rate of breathing |
| Inspiratory Time | Ti | | sec | Duration of inspiration |
| Expiratory Time | Te | | sec | Duration of expiration |
| End Inspiratory Pause | EIP | | msec | Pause between end of inspiration start of expiration |
| End Expiratory Pause | EEP | | msec | Pause between end of expiration and start of inspiration |
| Relaxation Time | RT | | sec | Decay of expiration to 36% maximum |
| Tidal Volume | TV | | ml | Volume of inspired air per breath |
| Minute Ventilation | MV = freq x TV | | ml/min | Total volume of air inspired per min |
| Peak Inspiratory Flow | PIF | | ml/sec | Maximum inspiratory flow |
| Peak Expiratory Flow | PEF | | ml/sec | Maximum expiratory flow |
| Expiratory flow at 50% | EF_50_ | | ml/sec | Expiratory flow at 50% expired TV |
| Non-eupneic breathing index | NEBI | | % | % of non-eupneic breaths per epoch |
| **B. Derived parameters** | | | | |
| Ti/Te | Ti/Te | | none | Inspiratory quotient |
| Inspiratory Drive | | TV/Ti | ml/sec | Central urge to inhale |
| Expiratory Drive | | TV/Te | ml/sec | Central drive to exhale |
| PIF/PEF | | PIF/PEF | none | Flow balance |
| Expiratory Time – Relaxation Time | | Te-RT | none | Expiratory delay |
| Apneic Pause (Te/RT)-1 | | Apneic Pause | none | Expiratory ratio |

**Supplementary Figure S1**

**Supplementary Figure S1.** Relationships between peak inspiratory flow (PIF), peak expiratory flow (PEF), relaxation time (RT) and expiratory time (Te).

**Supplementary Table S4.** Resting ventilatory parameters prior to administration of morphine
(10 mg/kg, IV) in the vehicle (saline) and L-CYSme groups of rats

| **Parameter** | **Vehicle** |  | **L-CYSme** |
| --- | --- | --- | --- |
| Group number | 6 |  | 6 |
| Age, days | 80.9 ± 0.4 |  | 80.8 ± 0.3 |
| Body weight, gram | 336 ± 2 |  | 335 ± 2 |
| Frequency, breaths/min | 107 ± 8 |  | 103 ± 5 |
| Tidal Volume (TV), ml | 2.42 ± 0.06 |  | 2.49 ± 0.05 |
| Minute Ventilation, ml/min | 257 ± 15 |  | 256 ± 11 |
| Inspiratory Time (Ti), sec | 0.230 ± 0.015 |  | 0.236 ± 0.005 |
| Expiratory Time (Te), sec | 0.366 ± 0.024 |  | 0.377 ± 0.016 |
| Inspiratory Time/Expiratory Time | 0.63 ± 0.01 |  | 0.63 ± 0.03 |
| End Inspiratory Pause, msec | 7.83 ± 0.33 |  | 7.90 ± 0.20 |
| End Expiratory Pause, msec | 34.6 ± 0.7 |  | 34.3 ± 0.3 |
| Peak Inspiratory Flow (PIF), ml/sec | 15.7 ± 0.7 |  | 15.9 ± 0.7 |
| Peak Expiratory Flow (PEF), ml/sec | 10.4 ± 0.5 |  | 10.5 ± 0.4 |
| PIF/PEF | 1.53 ± 0.09 |  | 1.52 ± 0.10 |
| Expiratory Flow at 50% TV, ml/sec | 0.44 ± 0.03 |  | 0.46 ± 0.04 |
| Relaxation Time (RT), sec | 0.21 ± 0.03 |  | 0.21 ± 0.02 |
| Apneic Pause, ((Te/RT)-1) | 1.80 ± 0.09 |  | 1.95 ± 0.16 |
| Inspiratory Drive (TV/Ti), ml/sec | 10.7 ± 0.5 |  | 10.7 ± 0.3 |
| Expiratory Drive (TV/Te), ml/sec | 6.74 ± 0.37 |  | 6.72 ± 0.32 |
| Non-Eupneic Breathing Index (NEBI), % | 3.41 ± 0.16 |  | 3.75 ± 0.39 |
| NEBI/Frequency, %/(breaths/min) | 3.3 ± 0.4 |  | 3.8 ± 0.5 |

The data are presented as mean ± SEM. There were 6 rats in each group. There were no between group differences in any parameter (*p* > 0.05, for all comparisons).

**Supplementary Table S5.** Total changes in ventilatory parameters that occurred over 15 minutes following the injection of morphine prior to the administration of vehicle (saline) or L-CYSme

| **Parameter** | **Vehicle** |  | **L-CYSme** |
| --- | --- | --- | --- |
| Frequency, % change | -9 ± 9 |  | -6 ± 9 |
| Tidal Volume (TV), % change | -40 ± 4* |  | -45 ± 2* |
| Minute Ventilation, % change | -45 ± 6* |  | -48 ± 5* |
| Inspiratory Time (Ti), % change | +34 ± 12* |  | +31 ± 3* |
| Expiratory Time (Te), % change | +12 ± 14 |  | +4 ± 11 |
| Ti/Te, % change | +32 ± 8* |  | +42 ± 13* |
| End Inspiratory Pause, % change | +118 ± 22* |  | +115 ± 11* |
| End Expiratory Pause, % change | +245 ± 13* |  | +255 ± 28* |
| Peak Inspiratory Flow (PIF), % change | -36 ± 5* |  | -34 ± 5* |
| Peak Expiratory Flow (PEF), % change | -10 ± 6 |  | +3 ± 5 |
| PIF/PEF, % change | -26 ± 9* |  | -34 ± 7* |
| Expiratory Flow at 50% TV, % change | -15 ± 10 |  | +13 ± 7 |
| Relaxation Time (RT), % change | -11 ± 9 |  | -9 ± 8 |
| Apneic Pause, ((Te/RT)-1), % change | +25 ± 7* |  | +31 ± 10* |
| Inspiratory Drive (TV/Ti), % change | -51 ± 5* |  | -55 ± 3* |
| Expiratory Drive (TV/Te), % change | -39 ± 6* |  | -40 ± 9* |
| Non-Eupneic Breathing Index (NEBI), % change | +82 ± 20* |  | +79 ± 20* |
| NEBI/Frequency, % change | +130 ± 22* |  | +119 ± 15* |

The data are presented as mean ± SEM. There were 6 rats in each group. **p* < 0.05, significant change from resting (Pre) values. There were no between group differences for any parameter (*p* > 0.05, for all comparisons).

**Supplementary Table S6.** Arithmetic changes in arterial blood chemistry values during various stages of the study

|  |  |  | **Arithmetic changes** | | | |
| --- | --- | --- | --- | --- | --- | --- |
| **Parameter** | **Group** | **Pre** | **M15:D0** | **M30:D15** | **M45:D30** | **M60:D45** |
| pH | Vehicle | 7.439 ± 0.011 | -0.234 ± 0.011 | -0.230 ± 0.013 | -0.205 ± 0.020 | -0.196 ± 0.019 |
|  | L-Cysteine | 7.438 ± 0.010 | -0.231 ± 0.014 | -0.228 ± 0.012 | -0.204 ± 0.007 | -0.182 ± 0.015 |
|  | L-CYSme | 7.436 ± 0.010 | -0.238 ± 0.013 | +0.028 ± 0.011 | +0.009 ± 0.009 | +0.003 ± 0.008 |
| pCO_2_, mmHg | Vehicle | 36.1 ± 0.4 | +21.9 ± 0.4* | +21.9 ± 0.7* | +21.5 ± 0.9* | +18.7 ± 0.8* |
|  | L-Cysteine | 36.4 ± 0.6 | +21.8 ± 0.5* | +21.5 ± 0.6* | +20.8 ± 0.6* | +18.8 ± 0.5* |
|  | L-CYSme | 35.9 ± 0.4 | +21.4 ± 0.7* | -1.6 ± 0.6^†^ | +0.4 ± 0.4^†^ | +0.5 ± 0.4^†^ |
| pO_2,_ mmHg | Vehicle | 93.9± 0.3 | -37.5 ± 0.5* | -37.0 ± 0.8* | -35.3 ± 0.8* | -32.5 ± 0.8* |
|  | L-Cysteine | 93.3 ± 0.5 | -37.4 ± 1.0* | -36.1 ± 1.3* | -34.4 ± 0.9* | -32.2 ± 0.9* |
|  | L-CYSme | 93.2 ± 0.4 | -35.5 ± 1.1* | +0.9 ± 0.3^†^ | +0.9 ± 0.4^†^ | +1.4 ± 0.4^†^ |
| sO_2,_ % | Vehicle | 99.8 ± 0.1 | -36.1 ± 1.0* | -34.2 ± 1.1* | -30.3 ± 1.2* | -26.8 ± 0.8* |
|  | L-Cysteine | 99.5 ± 0.2 | -35.7 ± 0.7* | -34.9 ± 0.7* | -30.8 ± 0.8* | -28.3 ± 1.0* |
|  | L-CYSme | 99.5 ± 0.2 | -37.1 ± 0.8* | +0.4 ± 0.2^†^ | +0.2 ± 0.2^†^ | +0.2 ± 0.3^†^ |
| A-gradient_,_ mmHg | Vehicle | 15.8 ± 0.4 | +13.2 ± 0.4* | 12.6 ± 0.8* | 11.4 ± 1.1* | 11.8 ± 1.1* |
|  | L-Cysteine | 16.0 ± 0.8 | +13.1 ± 1.2* | 12.2 ± 1.4* | 11.3 ± 1.3* | 11.7 ± 1.2* |
|  | L-CYSme | 16.6 ± 0.5 | +11.7 ± 1.3* | 0.8 ± 0.4^†^ | -1.4 ± 0.3*^,†^ | -2.0 ± 0.5*^,†^ |

L-CYSme, L-cysteine methyl ester. A-a gradient, Alveolar-arterial gradient. M15, M30, M45 and M60 refers to 15-, 30-, 45- and 60-min post-morphine injection. The terms D0, D15, D30 and D45 refer to 0-, 15-, 30- and 45-min post-vehicle, L-Cysteine or L-CYSme injection. There were 9 rats in each group. **p* < 0.05, significant change from Pre values. ^†^*p* < 0.05, significant change L-CYSme *versus* vehicle.

**Supplementary Table S7.** Summary of the changes in tail-flick and hot-plate latencies

|  |  |  | **Pre** |  | **Post-injection** |  | **Pre-morphine** |  | **Post-injection (%Pre)** |  | **Pre-morphine**  **(%Pre)** |
| --- | --- | --- | --- | --- | --- | --- | --- | --- | --- | --- | --- |
| TFL | Morphine 5 | Vehicle | 3.12 ± 0.11 |  | 3.12 ± 0.12 |  | 3.14 ± 0.12 |  | 0.1 ± 1.1 |  | +0.7 ± 1.0 |
|  |  | L-CYSme | 3.17 ± 0.14 |  | 3.44 ± 0.16 |  | 3.18 ± 0.13 |  | +8.8 ± 1.9*^,†^ |  | +0.1 ± 1.1 |
|  | Morphine 10 | Vehicle | 3.13 ± 0.13 |  | 3.16 ± 0.12 |  | 3.14 ± 0.13 |  | +0.8 ± 0.9 |  | +0.4 ± 1.0 |
|  |  | L-CYSme | 3.27 ± 0.13 |  | 3.77 ± 0.16 |  | 3.26 ± 0.15 |  | +12.4 ± 0.9*^,†^ |  | -0.3 ± 1.1 |
| HPL | Morphine 5 | Vehicle | 20.8 ± 0.5 |  | 20.7 ± 0.6 |  | 20.6 ± 0.5 |  | -0.2 ± 0.6 |  | -0.9 ± 0.5 |
|  |  | L-CYSme | 20.2 ± 0.5 |  | 23.2 ± 0.7 |  | 20.1 ± 0.5 |  | +15.2 ± 2.1*^,†^ |  | -0.2 ± 0.3 |
|  | Morphine 10 | Vehicle | 19.9 ± 0.6 |  | 19.8 ± 0.7 |  | 19.9 ± 0.6 |  | -0.4 ± 0.7 |  | +0.2 ± 0.4 |
|  |  | L-CYSme | 20.1 ± 0.4 |  | 23.6 ± 0.6 |  | 20.2 ± 0.4 |  | +17.3 ± 2.1*^,†^ |  | +0.5 ± 0.8 |
| TFL | Morphine 5 | Vehicle | 3.13 ± 0.11 |  | 3.14 ± 0.10 |  | 3.14 ± 0.12 |  | +0.5 ± 1.1 |  | +0.4 ± 1.7 |
|  |  | L-Cysteine | 3.23 ± 0.12 |  | 2.98 ± 0.14 |  | 3.18 ± 0.13 |  | -7.7 ± 0.8*^,†^ |  | -1.3 ± 1.7 |
|  | Morphine 10 | Vehicle | 3.08 0.11 |  | 3.13 ± 0.14 |  | 3.16 ± 0.13 |  | +1.2 ± 1.3 |  | +1.7 ± 1.1 |
|  |  | L-Cysteine | 3.28 0.13 |  | 2.85 ± 0.13 |  | 3.27 ± 0.14 |  | -13.2 ± 1.4*^,†^ |  | -0.2 ± 1.5 |

L-CYSme, L-cysteine methyl ester. TFL, tail-flick latency; HPL, hot-plate latency. There were 9 rats in the vehicle- or L-CYSme-injected groups and 6 rats in the L-Cysteine-injected group. **p*< 0.05, significant change from Pre values. ^†^ *p* < 0.05, significant change
L-CYSme *versus* vehicle.

**Supplementary Figure S2**

**A.**

**C.**

**B.**

**D.**

**Supplementary Figure S2.** Changes in tail-flick latency values elicited by intravenous injection of morphine at 5 mg/kg (**Panel A**) or 10 mg/kg (**Panel B**) in freely-moving rats that had received injections of vehicle (VEH, saline) or L-cysteine methyl ester (L-CYSme, 500 μmol/kg, IV) 20 minutes before-hand. **Panels C** and **D** display the data as maximum possible effect (%MPE). The data are shown as mean ± SEM. There were 9 rats in each group. **p* < 0.05, significant change from Pre. ^†^*p* < 0.05, significant change L-CYme *versus* vehicle.

**Supplementary Figure S3**

**B.**

**A.**

**C.**

**D.**

**Supplementary Figure S3.** Changes in hot-plate latency values elicited by intravenous injection of morphine at 5 mg/kg (**Panel A**) or 10 mg/kg (**Panel B**) in freely-moving rats that had received injections of vehicle (VEH, saline) or L-cysteine methyl ester (L-CYSme, 500 μmol/kg, IV) 20 minutes before-hand. **Panels C** and **D** display the data as maximum possible effect (%MPE). The data are shown as mean ± SEM. There were 9 rats in each group. **p* < 0.05, significant change from Pre. ^†^*p* < 0.05, significant change L-CYme *versus* vehicle.

**Supplementary Figure S4**

**A.**

**B.**

**D.**

**C.**

**Supplementary Figure S4.** Changes in tail-flick latency values elicited by intravenous injection of morphine at 5 mg/kg (**Panel A**) or 10 mg/kg (**Panel B**) in freely-moving rats that had received injections of vehicle (VEH, saline) or L-Cysteine (500 μmol/kg, IV) 20 minutes before-hand. **Panels C** and **D** display the data as maximum possible effect (%MPE). The data are shown as mean ± SEM. There were 9 rats in the vehicle group and 6 rats in the L-Cysteine group. **p* < 0.05, significant change from Pre.
